# Guideline-directed medical therapy use, persistence, and cardiovascular outcomes following ST-segment elevation myocardial infarction: a pooled analysis of three randomised trials with external registry validation

**DOI:** 10.1016/j.lanepe.2026.101919

**Published:** 2026-11-07

**Authors:** Daijiro Tomii, Dik Heg, Sylvain Losdat, Miklos Rohla, Jonas D. Häner, Lukas Hunziker, Monika Fürholz, Noé Corpataux, Jonas Lanz, Fabien Praz, Olivier Muller, Juan F. Iglesias, Stephan Windecker, Lorenz Räber, Thomas Pilgrim

**Affiliations:** a Department of Cardiology, Bern University Hospital, Inselspital, University of Bern, Bern, Switzerland; b Department of Clinical Research, University of Bern, Bern, Switzerland; c Department of Cardiology, Lausanne University Hospital, Lausanne, Switzerland; d Department of Cardiology, Geneva University Hospital, Geneva, Switzerland

**Keywords:** Acute coronary syndrome, Guideline-directed medical therapy, Percutaneous coronary intervention, ST-Segment elevation myocardial infarction

## Abstract

**Background:**

Implementation and long-term persistence of guideline-directed medical therapy (GDMT) following ST-segment elevation myocardial infarction (STEMI) remain suboptimal. We assessed the implementation and persistence of four-class GDMT after STEMI and its association with outcomes.

**Methods:**

We performed a patient-level pooled analysis of three randomised trials of patients undergoing percutaneous coronary intervention for STEMI. GDMT was defined by the number of drug classes prescribed (0–4). External validation was conducted using a real-world PCI registry.

**Findings:**

The study included 2270 patients from the randomised trials and 5643 from a real-world PCI registry. More than two-thirds of patients received all four GDMT classes at discharge (ranging from 66.9% [398/595] to 83.5% [339/406]), but this proportion declined over 5 years (ranging from 40.5% [408/1008] to 53.1% [276/520]). In exploratory analyses, higher GDMT class at discharge was associated with a lower risk of the composite of cardiovascular death, stroke, or myocardial infarction at 5 years (adjusted HR [95% CI] for class 4 versus 0–2: 0.49 [0.33–0.72]; class 3 versus 0–2: 0.49 [0.28–0.85]). In a time-dependent analysis, higher GDMT class during follow-up was associated with a lower risk of the composite outcome (adjusted IRR [95% CI] for class 4 versus 0–2: 0.71 [0.52–0.95]; class 3 versus 0–2: 0.59 [0.41–0.86]). Similar longitudinal patterns were observed in the real-world PCI registry.

**Interpretation:**

Although the majority of patients received all four GDMT classes at discharge after STEMI, this proportion declined during long-term follow-up. In exploratory analyses, higher GDMT class was associated with a lower risk of cardiovascular events.

**Funding:**

No specific funding was received for the present study. Funding for the individual trials and registry is reported in the Acknowledgements.

**Research in context**

**Evidence before this study**

We searched PubMed from database inception to March 1, 2026, using the search terms (“acute myocardial infarction” OR “myocardial infarction” OR “STEMI” OR “ST-segment elevation myocardial infarction”) AND (“guideline-directed medical therapy” OR “optimal medical therapy”). No language restrictions were applied. We considered original clinical studies evaluating the implementation or persistence of combinations of recommended pharmacological secondary prevention therapies after acute myocardial infarction. We identified four relevant studies. Previous evidence was predominantly derived from observational studies, which consistently showed that both implementation at discharge and persistence during follow-up of recommended pharmacological therapy after acute myocardial infarction remain suboptimal. However, previous studies have predominantly included heterogeneous populations with acute coronary syndrome or acute myocardial infarction, with limited longitudinal data specifically characterising the implementation and long-term persistence of established pharmacological therapy after STEMI. Against a background of plateauing reductions in STEMI-related mortality despite advances in reperfusion therapy, further characterisation of long-term GDMT patterns in a well-defined STEMI population is warranted.

**Added value of this study**

This study provides long-term, STEMI-specific patient-level data on the implementation and persistence of four-class GDMT across successive treatment eras, using data from three investigator-initiated randomised multicentre trials with external validation in a contemporary real-world PCI registry. More than two-thirds of patients received all four GDMT classes at hospital discharge, but this proportion declined during long-term follow-up, with broadly consistent patterns observed across the randomised trial and real-world populations. In exploratory analyses, higher GDMT class at discharge and during follow-up was associated with a lower risk of cardiovascular death, stroke, or myocardial infarction.

**Implications of all the available evidence**

The observed decline in four-class GDMT over time is an important consideration as secondary-prevention strategies after STEMI continue to evolve with emerging evidence and new therapeutic approaches. Alongside ongoing efforts to define the optimal composition of contemporary secondary prevention, the present findings highlight persistent challenges in the long-term implementation and persistence of established pharmacological therapies. More broadly, the residual burden of STEMI underscores the continued importance of identifying and managing cardiovascular risk before myocardial infarction occurs.

## Introduction

Over the past decades, advances in early reperfusion strategies, particularly the widespread adoption of primary percutaneous coronary intervention (PCI), have reduced mortality in patients with ST-segment elevation myocardial infarction (STEMI).^1^, ^2^ However, these improvements have been primarily observed in early mortality rates, and long-term survival after STEMI does not fully normalise to that of the general population, even after successful revascularisation.^3^, ^4^ Secondary prevention is a cornerstone of lifelong management after STEMI, with the aim of reducing the risk of recurrent cardiovascular events and improving survival and patient-reported health status. In this context, guideline-directed medical therapy (GDMT) plays a key role supported by robust clinical evidence.^5^, ^6^ Nevertheless, several studies have suggested that the implementation and long-term persistence of GDMT after acute myocardial infarction remain suboptimal.^7^, ^8^, ^9^ Previous studies have predominantly included broader populations with acute coronary syndrome or acute myocardial infarction, with limited long-term patient-level data specifically characterising established pharmacological therapy after STEMI across different treatment eras. Differences in study populations and follow-up periods may further complicate comparisons of treatment patterns over time. Given the plateau in STEMI-related mortality reductions in the contemporary era, a renewed evaluation of the implementation and long-term persistence of GDMT in a well-defined STEMI population is warranted. Accordingly, the present study systematically assessed the implementation and long-term persistence of four-class GDMT after STEMI using data from investigator-initiated randomised trials conducted across different treatment eras. To assess the consistency of these findings in routine clinical practice, the findings were externally validated using longitudinal data from a prospective real-world PCI registry.

## Methods

### Study design and cohort

We performed a patient-level pooled observational analysis using data from three investigator-initiated, prospective, multicentre, randomised trials: COMFORTABLE-AMI (Comparison of Biolimus Eluted From an Erodible Stent Coating With Bare Metal Stents in Acute ST-Elevation Myocardial Infarction), BIOSCIENCE (Sirolimus-eluting Stents With Biodegradable Polymer Versus an Everolimus-eluting Stents), and BIOSTEMI (A Comparison of an Ultrathin Strut Biodegradable Polymer Sirolimus-Eluting Stent With a Durable Polymer Everolimus-Eluting Stent for Patients With Acute ST-Segment Elevation Myocardial Infarction Undergoing Primary Percutaneous Coronary Intervention). These trials were selected at the study-planning stage because they were investigator-initiated trials with available individual patient-level data, STEMI and clinical endpoints were defined and assessed using broadly consistent criteria, and 5-year clinical follow-up had been completed. These characteristics allowed longitudinal assessment of GDMT using a common analytical framework. The present study used the trial data observationally rather than according to the original randomised treatment allocation. The randomised interventions concerned coronary stent strategies rather than GDMT. Prespecified eligibility criteria for the present analysis included STEMI and PCI with drug-eluting stents (DES). Patients meeting these eligibility criteria were pooled irrespective of their original treatment allocation. The rationale and design, as well as details on study conduct, randomisation, masking, data management, data monitoring, and results of each trial have been reported previously.^10^, ^11^, ^12^, ^13^, ^14^, ^15^ For the purpose of this study, patients treated with non-DES PCI, those without STEMI (only applicable to BIOSCIENCE), those with missing information on GDMT prescription at discharge after STEMI, or patients with in-hospital death were excluded.

The CARDIOBASE Bern PCI registry is a prospective, single-centre registry that collects baseline clinical and procedural data as well as clinical outcomes of all patients undergoing PCI at Bern University Hospital (ClinicalTrials.gov Identifier: NCT02241291). Details of the rationale and design of the registry have been described previously.^16^ Patients with STEMI who underwent PCI with DES between 2009 and 2022 and had available information on GDMT prescription at discharge after PCI were included in the present analysis.

The present study adhered to the Strengthening the Reporting of Observational Studies in Epidemiology guidelines.^17^

### Data collection, definition, and outcomes of interest

Details of the data collection and patient follow-up of the randomised trials and the registry have been published elsewhere.^10^, ^11^, ^12^, ^13^, ^14^, ^15^, ^16^ In brief, all baseline clinical, medication, procedural, and follow-up data were prospectively recorded and stored in a dedicated database, held at the Clinical Trials Unit at the University of Bern, Switzerland. For the three randomised trials, electronic case report forms were developed within the same shared electronic data capture environment, with consistent variable naming maintained across trials. Variables required for the present analysis were aligned across trials, and individual patient-level data were combined into a common analytical dataset. Clinical events were based on the original adjudication in each trial and were not re-adjudicated for the present analysis. All three randomised trials completed 5 years of clinical follow-up after the index PCI, while 1-year follow-up data were available in the real-world PCI registry. All clinical endpoints in the randomised trials and the registry were adjudicated by an independent clinical event committee. Specific information on study period, key inclusion and exclusion criteria, and definitions of STEMI and clinical endpoints in each randomised trial is summarised in the Supplementary Information.

For the purpose of the present study, four-class GDMT was defined as a combination of any antithrombotic therapy (aspirin, P2Y12 inhibitors, or oral anticoagulants), lipid-lowering therapy (statins or other lipid-lowering therapies), renin-angiotensin system inhibitors (angiotensin-converting enzyme inhibitors, angiotensin receptor blockers, or angiotensin receptor-neprilysin inhibitors) and beta-blockers, and categorised according to the number of drug classes prescribed (GDMT class 0–4). This four-class definition was selected to provide a consistent framework across the treatment eras represented by the study cohorts and was not intended to encompass all components of contemporary secondary prevention or to imply that all four drug classes were uniformly indicated in every patient. Consistent with clinical guidelines supporting consideration of renin-angiotensin system inhibitors and beta-blockers after acute coronary syndrome irrespective of left ventricular ejection fraction, these therapies were included within the four-class GDMT framework without modification according to left ventricular systolic function.^5^, ^6^

The primary objective of the present study was to describe the implementation and persistence of GDMT after STEMI. In exploratory analyses, we investigated the association between GDMT prescription and clinical outcomes, including a composite of cardiovascular death, stroke, or myocardial infarction, individual components of the composite outcome, and all-cause death. GDMT prescription patterns were analysed separately for each randomised trial, while clinical outcomes were assessed in the pooled randomised cohort. The real-world PCI registry data served as an independent cohort for external validation of the findings from the pooled randomised trials.

### Statistical analysis

Categorical variables are presented as frequencies and percentages and were compared using the chi-square test or Fisher's exact test, as appropriate. Continuous variables are presented as mean ± standard deviation (SD) and were compared between groups using analysis of variance (ANOVA). Prescription rates were calculated among patients who were alive and had available medication information at each follow-up time point. We also evaluated GDMT status using the population at discharge as a fixed denominator, with death and unavailable medication information included as separate categories. No imputation was performed for missing data. The median calendar year corresponding to each follow-up time point (discharge, 1 year, 2 years, and 5 years) was calculated for each randomised trial. Year-specific GDMT prescription rates at discharge and 1 year in the real-world PCI registry were also provided. Time-to-event curves were constructed using the Kaplan–Meier method. Time to event was calculated from discharge to the first occurrence of the respective clinical endpoint. Patients without the respective event were censored at death when death was not part of the endpoint, at the last valid contact in cases of withdrawal of consent or loss to follow-up, or administratively at 5 years. Cox proportional hazards models were used to calculate hazard ratios (HRs) and 95% confidence intervals (95% CIs), and statistical significance was assessed using Wald tests. For the Cox analyses, GDMT class at discharge was treated as a fixed baseline exposure, and only the first occurrence of each event type was considered. The proportional hazards assumption was assessed using Schoenfeld residuals. When evidence of non-proportional hazards was observed, additional piecewise Cox analyses were performed. Multivariable adjustment was performed using prespecified baseline variables considered clinically relevant or potentially associated with outcomes: age, female sex, body mass index, Killip class III or IV, diabetes, chronic kidney disease, atrial fibrillation, peripheral artery disease, and chronic obstructive pulmonary disease. For the pooled randomised cohort, standard errors were adjusted for clustering by trial ID using cluster-robust variance estimation. In an additional exploratory analysis, we examined the association between specific pharmacological combinations comprising GDMT class 3 at discharge and the composite cardiovascular outcome during follow-up, using GDMT class 4 as the reference group and the same multivariable adjustment as the primary Cox analysis. Subgroup analyses were performed according to age (<75 or ≥75 years), sex, Killip class (I-II or III-IV), chronic kidney disease, diabetes mellitus, atrial fibrillation, chronic obstructive pulmonary disease, and left ventricular ejection fraction (<40%, 40–50%, or ≥50%). Since in-hospital cardiovascular events (stroke and myocardial infarction) may influence the prescription of GDMT, a sensitivity analysis including only post-discharge events was performed. Given that prescription status can change over time, an analysis incorporating GDMT class as a time-dependent variable was performed. The overall follow-up period was the same as for the Cox analyses, but follow-up time was divided into exposure periods according to the dates at which GDMT class was updated. GDMT class was updated at 1 and 2 years if it differed from the previous period. All post-discharge occurrences of each event type were included and attributed to the corresponding GDMT class period, with total observation time expressed in person-years. Incidence rates are expressed per 100 person-years. Poisson regression models with log(person-time) as an offset were used to estimate incidence rate ratios (IRRs) with 95% CIs, and statistical significance was assessed using Wald tests. For the unadjusted Poisson models, standard errors were adjusted for clustering by patient ID using cluster-robust variance estimation. Multivariable Poisson regression models were adjusted for age, female sex, body mass index, Killip class III or IV, diabetes, chronic kidney disease, atrial fibrillation, peripheral artery disease, and chronic obstructive pulmonary disease, with a random effect for patient ID to account for within-patient correlation. Overdispersion was assessed using a deviance goodness-of-fit test and did not materially affect the Poisson model estimates. All statistical analyses were two-sided and performed using Stata 18 (StataCorp LLC), with P < 0.05 considered statistically significant.

### Ethics approval

The three randomised trials were approved by the institutional review board or ethics committee at each participating site and were conducted in accordance with the principles of the Declaration of Helsinki. All patients provided written informed consent before enrolment.^10^, ^11^, ^12^, ^13^, ^14^, ^15^

The CARDIOBASE Bern PCI registry is approved by the Cantonal Ethics Committee in Bern (KEK 137/14), and all patients provided written informed consent.

### Role of the funding source

The funders of the trials or the registry had no role in the design and conduct of the study; collection, management, analysis, and interpretation of the data; preparation, review, or approval of the manuscript; and the decision to submit the manuscript for publication.

## Results

### Study population

A total of 2270 STEMI patients from three randomised trials conducted between 2009 and 2018, with a completion period between 2016 and 2023, and 5643 patients from the real-world registry of patients undergoing PCI between 2009 and 2022 were included in the present analysis (Supplementary Figure S1). The mean age and the proportion of male patients were similar between the pooled randomised cohort (61 ± 11 years; 78.3% male [1777/2270] and 21.7% female [493/2270]) and the real-world PCI registry (64 ± 12 years; 76.9% male [4339/5643] and 23.1% female [1304/5643]), while a higher prevalence of Killip class III or IV and a greater comorbidity burden were observed in the real-world PCI registry.

### Implementation and continuation of GDMT

In the randomised trials, the majority of patients were prescribed 4 classes of GDMT medications (GDMT class 4) at discharge, ranging from 66.9% (398/595) to 83.5% (339/406). Even among patients who did not receive GDMT class 4, GDMT class 3 was the most common, whereas GDMT class 2 or lower was observed in relatively few patients (23/595, 7/406, and 55/1269, respectively). During follow-up, the proportion of patients receiving GDMT class 4 declined consistently across all trials, with proportions ranging from 40.5% (408/1008) to 53.1% (276/520) at 5 years. In contrast, the proportion of patients receiving GDMT class 2 or lower increased over time, reaching 13.1% (68/520) to 23.7% (239/1008) at 5 years (Fig. 1 and Supplementary Table S1). A consistent decline in GDMT class 4 was observed when the population at discharge was used as a fixed denominator, with death and unavailable medication information included as separate categories (Supplementary Table S2).

**Fig. 1:**
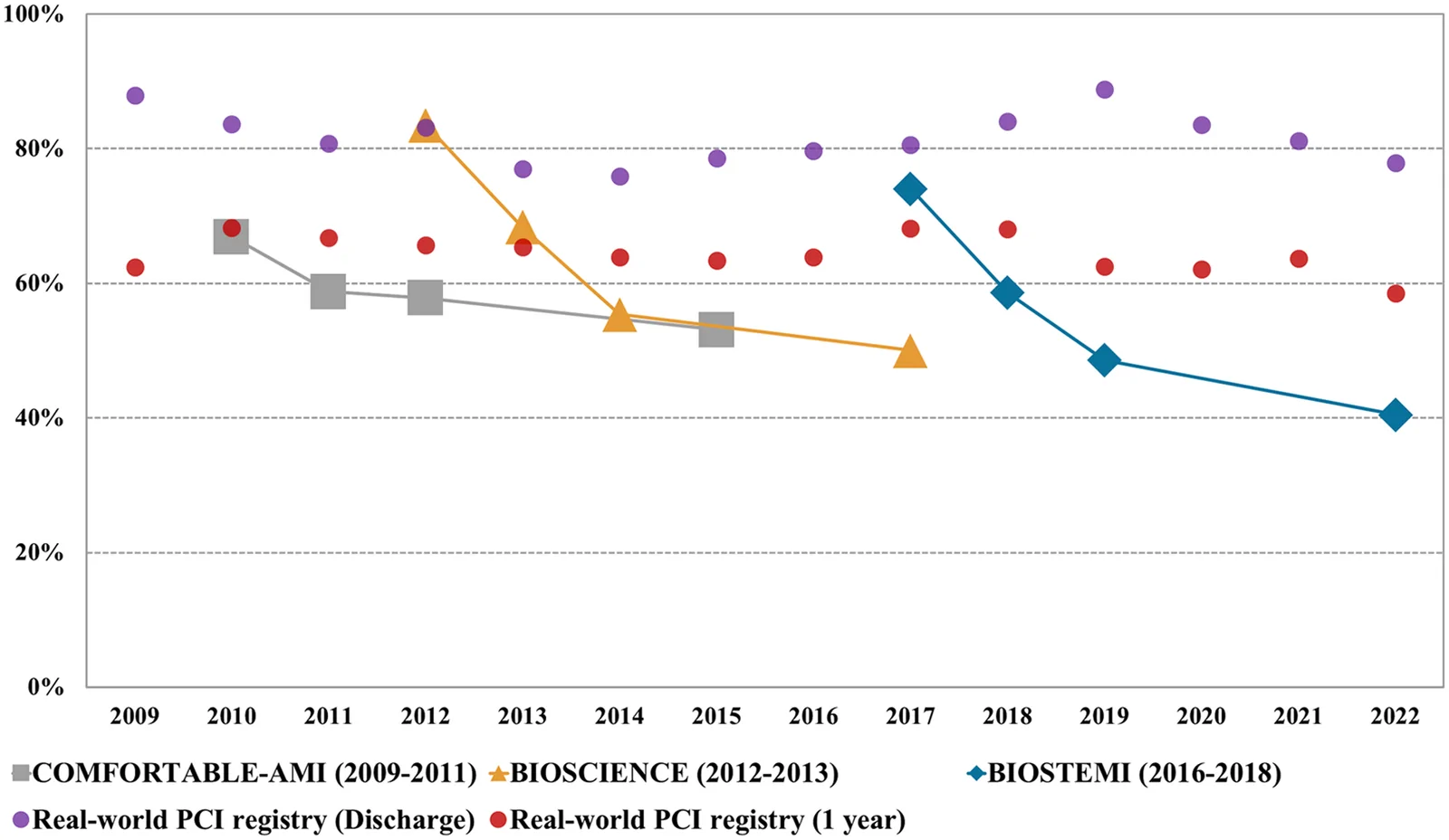
**GDMT class 4 prescription rates after STEMI over time.** GDMT class 4 prescription rates at each follow-up time point in the randomised trials (**solid lines with markers**) and corresponding temporal trends in the real-world PCI registry at discharge and 1-year follow-up (**circles**). Prescription rates were calculated among patients who were alive and had available medication information at each respective time point. For each randomised trial, the median calendar year corresponding to each follow-up visit is presented. GDMT, guideline-directed medical therapy; PCI, percutaneous coronary intervention; STEMI, ST-segment elevation myocardial infarction.

Similar results were observed in the real-world PCI registry across calendar years. At discharge, more than three-quarters of patients received GDMT class 4 (ranging from 75.9% [302/398] to 87.9% [255/290]), whereas the proportion of patients with GDMT class 0–2 was low. At 1 year, the annual proportion of patients receiving GDMT class 4 was lower than at discharge, ranging from 58.5% (127/217) to 68.3% (170/249), with correspondingly higher proportions of patients receiving GDMT class 3 and GDMT class 0–2 (Fig. 1 and Supplementary Tables S1–S3).

### GDMT components

Fig. 2 and Supplementary Table S4 summarise the prescription rates of individual GDMT components across study cohorts over time. Across the randomised trials, the use of any antithrombotic therapy remained consistently high throughout follow-up. Lipid-lowering therapy was also widely prescribed at discharge and largely maintained during follow-up; however, distinct temporal changes were observed within this category, characterised by a decline in statin use accompanied by an increase in the use of other lipid-lowering therapies. Renin-angiotensin system inhibitors and beta-blockers were frequently prescribed at discharge in the trials, ranging from 78.1% (464/594) to 94.3% (383/406) and from 80.2% (1018/1269) to 89.7% (364/406), respectively. However, a steady decline in the prescription rates was observed for both GDMT components over the five-year follow-up period across trials. These patterns were largely consistent with those observed in the real-world PCI registry, with prescription rates for each GDMT component falling within similar ranges.

**Fig. 2:**
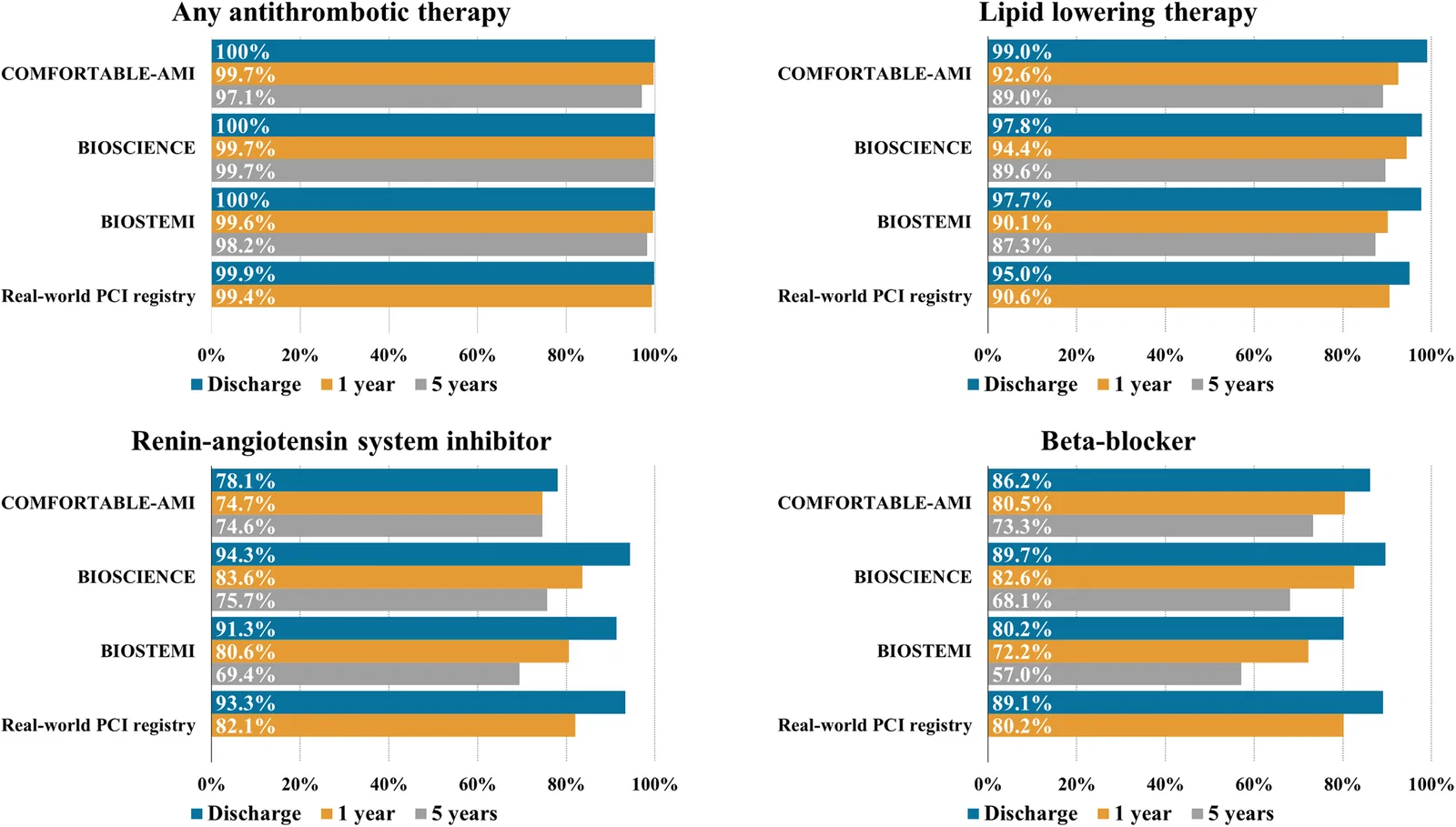
**Prescription rates of GDMT components after STEMI. Bar graphs** show prescription rates of individual GDMT components at each follow-up time point in the randomised trials and the real-world PCI registry. Abbreviations as in Fig. 1.

### Baseline patient demographics and PCI characteristics according to GDMT class

Baseline patient characteristics stratified by GDMT class at discharge in the pooled randomised cohort and the real-world PCI registry are summarised in Table 1. The distribution of GDMT class at discharge was as follows: in the pooled randomised cohort, 1677/2270 patients (73.9%) had GDMT class 4, 508/2270 (22.4%) had class 3, and 85/2270 (3.7%) had class 0–2; in the real-world PCI registry, 4606/5643 patients (81.6%) had GDMT class 4, 830/5643 (14.7%) had class 3, and 207/5643 (3.7%) had class 0–2 at discharge, respectively. Higher GDMT class was associated with a higher proportion of male patients and higher body mass index. In contrast, patients with lower GDMT had a higher prevalence of Killip class III or IV and a higher burden of comorbidities, as well as left ventricular ejection fraction <40%, than those with higher GDMT class. These differences were more evident in the real-world PCI registry. In both cohorts, the use of statin or other lipid lowering therapy remained 15–20% without no statistically significant difference across GMDT classes at baseline.

**Table 1:** Patient demographics according to GDMT status at discharge.

	Pooled randomised cohort					Real-world PCI registry				
	All patients N = 2270	GDMT 4 class N = 1677	GDMT 3 class N = 508	GDMT 0–2 class N = 85	P value	All patients N = 5643	GDMT 4 class N = 4606	GDMT 3 class N = 830	GDMT 0–2 class N = 207	P value
Age, years	61 ± 11	61 ± 11	61 ± 12	63 ± 12	0.343	64 ± 12	63 ± 12	67 ± 13	71 ± 13	<0.001
Sex					0.001					<0.001
Female, n (%)	493 (21.7%)	334 (19.9%)	133 (26.2%)	26 (30.6%)		1304 (23.1%)	979 (21.3%)	254 (30.6%)	72 (34.3%)	
Male, n (%)	1777 (78.3%)	1343 (80.1%)	375 (73.8%)	59 (69.4%)		4339 (76.9%)	3627 (78.7%)	576 (69.4%)	136 (65.7%)	
Body mass index, kg/cm^2^	27.0 ± 4.3	27.1 ± 4.4	26.6 ± 4.1	26.2 ± 4.7	0.019	27.0 ± 4.5	27.1 ± 4.5	26.6 ± 4.5	26.3 ± 4.8	0.002
Killip class III or IV, n (%)	60 (2.6%)	38 (2.3%)	15 (3.0%)	7 (8.2%)	0.003	642 (11.4%)	435 (9.5%)	143 (17.2%)	64 (31.1%)	<0.001
Medical history										
Hypertension, n (%)	1042 (46.0%)	795 (47.5%)	219 (43.2%)	28 (33.3%)	0.014	2914 (51.9%)	2353 (51.2%)	464 (56.4%)	97 (48.5%)	0.014
Hypercholesterolaemia, n (%)	1143 (50.6%)	853 (51.0%)	257 (51.1%)	33 (39.8%)	0.130	2592 (46.3%)	2155 (47.1%)	364 (44.4%)	73 (36.7%)	0.008
Diabetes mellitus, n (%)	288 (12.7%)	220 (13.1%)	59 (11.6%)	9 (10.6%)	0.563	847 (15.0%)	650 (14.1%)	156 (18.9%)	41 (20.3%)	<0.001
Active smoker, n (%)	994 (44.5%)	732 (44.4%)	223 (44.4%)	39 (46.4%)	0.936	2119 (38.1%)	1798 (39.5%)	266 (32.8%)	55 (27.9%)	<0.001
Chronic renal failure (eGFR <60 mL/min), n (%)	215 (9.8%)	156 (9.6%)	49 (10.0%)	10 (12.5%)	0.692	942 (19.5%)	671 (16.9%)	203 (29.0%)	68 (45.0%)	<0.001
Previous PCI, n (%)	101 (4.4%)	67 (4.0%)	27 (5.3%)	7 (8.2%)	0.102	484 (8.6%)	397 (8.6%)	76 (9.2%)	11 (5.4%)	0.231
Previous CABG, n (%)	25 (1.1%)	20 (1.2%)	4 (0.8%)	1 (1.2%)	0.744	114 (2.0%)	85 (1.8%)	22 (2.7%)	7 (3.5%)	0.104
Previous myocardial infarction, n (%)	100 (4.4%)	70 (4.2%)	24 (4.7%)	6 (7.1%)	0.415	401 (7.1%)	326 (7.1%)	60 (7.3%)	15 (7.4%)	0.971
Previous CVE, n (%)	16 (2.7%)	11 (2.8%)	5 (2.9%)	0 (0%)	0.717	206 (3.7%)	149 (3.2%)	42 (5.1%)	15 (7.4%)	0.001
Atrial fibrillation, n (%)	39 (2.3%)	29 (2.3%)	6 (1.8%)	4 (6.5%)	0.079	214 (4.6%)	149 (4.0%)	47 (6.7%)	18 (10.3%)	<0.001
Peripheral artery disease, n (%)	50 (2.2%)	33 (2.0%)	9 (1.8%)	8 (9.4%)	<0.001	185 (3.3%)	140 (3.0%)	35 (4.2%)	10 (5.0%)	0.082
COPD, n (%)	29 (1.7%)	23 (1.8%)	3 (0.9%)	3 (4.8%)	0.086	197 (3.5%)	154 (3.4%)	30 (3.6%)	13 (6.4%)	0.064
Echocardiography	N = 1664	n = 1234	n = 372	n = 58		N = 5293	n = 4346	n = 766	n = 181	
Left ventricular ejection fraction at baseline, %	48.9 ± 10.9	48.1 ± 10.5	51.2 ± 11.3	50.2 ± 13.0	<0.001	45.1 ± 11.5	45.0 ± 11.2	45.8 ± 12.5	43.4 ± 13.6	0.024
<40%, n (%)	254 (15.3%)	198 (16.0%)	46 (12.4%)	10 (17.2%)	0.205	1330 (25.1%)	1070 (24.6%)	199 (26.0%)	61 (33.7%)	0.019
40–50%, n (%)	528 (31.7%)	428 (34.7%)	89 (23.9%)	11 (19.0%)	<0.001	1767 (33.4%)	1519 (35.0%)	204 (26.6%)	44 (24.3%)	<0.001
≥50%, n (%)	882 (53.0%)	608 (49.3%)	237 (63.7%)	37 (63.8%)	<0.001	2196 (41.5%)	1757 (40.4%)	363 (47.4%)	76 (42.0%)	0.001
Baseline medication										
Antithrombotic therapy, n(%)	351 (16.0%)	249 (15.4%)	85 (17.1%)	17 (20.7%)	0.324	1202 (23.8%)	915 (22.0%)	223 (29.9%)	64 (40.8%)	<0.001
Aspirin, n (%)	303 (13.8%)	217 (13.4%)	72 (14.5%)	14 (17.3%)	0.537	960 (19.0%)	743 (17.9%)	169 (22.7%)	48 (30.8%)	<0.001
P2Y12 inhibitor, n (%)	42 (1.9%)	34 (2.1%)	7 (1.4%)	1 (1.2%)	0.549	189 (3.7%)	149 (3.6%)	30 (4.0%)	10 (6.4%)	0.176
Oral anticoagulant, n (%)	42 (1.9%)	28 (1.7%)	12 (2.4%)	2 (2.5%)	0.589	219 (4.3%)	150 (3.6%)	55 (7.4%)	14 (9.0%)	<0.001
Statin or other lipid lowering therapy, n (%)	342 (15.6%)	258 (16.0%)	73 (14.7%)	11 (13.8%)	0.688	983 (19.5%)	798 (19.2%)	158 (21.2%)	27 (17.4%)	0.362
Statin, n (%)	326 (14.9%)	248 (15.4%)	67 (13.5%)	11 (13.8%)	0.547	957 (19.0%)	775 (18.7%)	155 (20.8%)	27 (17.4%)	0.343
Other lipid lowering therapy, n (%)	25 (1.1%)	14 (0.9%)	10 (2.0%)	1 (1.2%)	0.111	92 (1.8%)	77 (1.9%)	14 (1.9%)	1 (0.6%)	0.537
Renin-angiotensin system inhibitor, n (%)	519 (23.8%)	403 (25.1%)	107 (21.6%)	9 (11.2%)	0.007	1433 (28.4%)	1167 (28.2%)	231 (31.1%)	35 (22.6%)	0.067
Beta-blocker, n (%)	315 (14.4%)	255 (15.9%)	53 (10.7%)	7 (8.8%)	0.005	923 (18.3%)	764 (18.4%)	136 (18.3%)	23 (14.8%)	0.522
Discharge medication										
Antithrombotic therapy, n(%)	2270 (100%)	1677 (100%)	508 (100%)	85 (100%)	.	5637 (99.9%)	4606 (100%)	829 (99.9%)	202 (97.6%)	<0.001
Aspirin, n (%)	2264 (99.7%)	1673 (99.8%)	506 (99.6%)	85 (100%)	0.744	5597 (99.2%)	4579 (99.4%)	818 (98.6%)	200 (96.6%)	<0.001
P2Y12 inhibitor, n (%)	2263 (99.7%)	1674 (99.8%)	506 (99.6%)	83 (97.6%)	0.002	5607 (99.4%)	4584 (99.5%)	824 (99.3%)	199 (96.1%)	<0.001
Oral anticoagulant, n (%)	110 (4.8%)	82 (4.9%)	23 (4.5%)	5 (5.9%)	0.854	416 (7.4%)	327 (7.1%)	74 (8.9%)	15 (7.3%)	0.183
Statin or other lipid lowering therapy, n (%)	2225 (98.1%)	1677 (100%)	476 (93.7%)	72 (85.7%)	<0.001	5360 (95.0%)	4606 (100%)	646 (77.8%)	108 (52.4%)	<0.001
Statin, n (%)	2212 (97.5%)	1670 (99.6%)	471 (92.7%)	71 (84.5%)	<0.001	5348 (94.8%)	4595 (99.8%)	645 (77.7%)	108 (52.4%)	<0.001
Other lipid lowering therapy, n (%)	81 (3.6%)	62 (3.7%)	16 (3.1%)	3 (3.6%)	0.844	151 (2.7%)	132 (2.9%)	17 (2.0%)	2 (1.0%)	0.123
Renin-angiotensin system inhibitor, n (%)	2006 (88.4%)	1677 (100%)	325 (64.0%)	4 (4.8%)	<0.001	5265 (93.3%)	4606 (100%)	623 (75.1%)	36 (17.5%)	<0.001
Beta-blocker, n (%)	1894 (83.5%)	1677 (100%)	215 (42.3%)	2 (2.4%)	<0.001	5029 (89.1%)	4606 (100%)	392 (47.2%)	31 (15.0%)	<0.001
PCI characteristics										
Target lesion coronary artery/arteries, n (%)										
Left main artery	30 (1.3%)	24 (1.4%)	6 (1.2%)	0 (0%)	0.504	173 (3.1%)	128 (2.8%)	35 (4.2%)	10 (4.8%)	0.028
Left anterior descending artery	1010 (44.5%)	798 (47.6%)	184 (36.2%)	28 (32.9%)	<0.001	2862 (50.7%)	2397 (52.0%)	375 (45.2%)	90 (43.5%)	<0.001
Left circumflex artery	439 (19.3%)	330 (19.7%)	93 (18.3%)	16 (18.8%)	0.785	1223 (21.7%)	1001 (21.7%)	180 (21.7%)	42 (20.3%)	0.886
Right coronary artery	979 (43.1%)	675 (40.3%)	258 (50.8%)	46 (54.1%)	<0.001	2279 (40.4%)	1791 (38.9%)	392 (47.2%)	96 (46.4%)	<0.001
Graft	7 (0.3%)	6 (0.4%)	1 (0.2%)	0 (0%)	0.740	42 (0.7%)	31 (0.7%)	8 (1.0%)	3 (1.4%)	0.325
Number of lesions with PCI per patient, n (%)					0.028					0.635
1	1811 (79.8%)	1315 (78.4%)	429 (84.4%)	67 (78.8%)	0.012	3809 (67.5%)	3111 (67.5%)	555 (66.9%)	143 (69.1%)	0.822
2	357 (15.7%)	283 (16.9%)	58 (11.4%)	16 (18.8%)	0.009	1327 (23.5%)	1088 (23.6%)	190 (22.9%)	49 (23.7%)	0.900
≥3	102 (4.5%)	79 (4.7%)	21 (4.1%)	2 (2.4%)	0.537	507 (9.0%)	407 (8.8%)	85 (10.2%)	15 (7.2%)	0.288
Number of lesions stented per patient, n (%)					0.040					0.687
1	1844 (81.2%)	1341 (80.0%)	435 (85.6%)	68 (80.0%)	0.016	3964 (70.2%)	3241 (70.4%)	572 (68.9%)	151 (72.9%)	0.483
2	334 (14.7%)	265 (15.8%)	54 (10.6%)	15 (17.6%)	0.012	1241 (22.0%)	1014 (22.0%)	185 (22.3%)	42 (20.3%)	0.821
≥3	92 (4.1%)	71 (4.2%)	19 (3.7%)	2 (2.4%)	0.637	438 (7.8%)	351 (7.6%)	73 (8.8%)	14 (6.8%)	0.437
Total number of stents implanted across all lesions, n	1.69 ± 0.98	1.71 ± 1.00	1.59 ± 0.93	1.68 ± 0.95	0.057	1.97 ± 1.15	1.95 ± 1.13	2.10 ± 1.22	1.91 ± 1.20	0.002
Total stent length across all lesions, mm	37.4 ± 23.8	38.2 ± 24.2	34.9 ± 22.7	37.4 ± 21.6	0.022	45.5 ± 28.1	45.1 ± 27.9	48.4 ± 28.9	43.9 ± 30.2	0.004
Minimum stent diameter across all lesions, mm	3.03 ± 0.50	3.02 ± 0.50	3.07 ± 0.50	3.09 ± 0.46	0.046	2.89 ± 0.53	2.90 ± 0.53	2.87 ± 0.52	2.90 ± 0.55	0.404
Maximum stent diameter across all lesions, mm	3.26 ± 0.46	3.26 ± 0.46	3.26 ± 0.46	3.29 ± 0.44	0.813	3.22 ± 0.50	3.23 ± 0.50	3.21 ± 0.49	3.18 ± 0.55	0.430
Lesion with worst Pre-PCI TIMI flow, n (%)					0.040					0.070
0 or 1	1547 (68.4%)	1161 (69.5%)	332 (65.5%)	54 (64.3%)	0.163	4073 (72.5%)	3345 (72.9%)	585 (70.7%)	143 (69.1%)	0.216
2	314 (13.9%)	238 (14.3%)	69 (13.6%)	7 (8.3%)	0.304	766 (13.6%)	624 (13.6%)	119 (14.4%)	23 (11.1%)	0.471
3	399 (17.6%)	270 (16.2%)	106 (20.9%)	23 (27.4%)	0.003	782 (13.9%)	617 (13.5%)	124 (15.0%)	41 (19.8%)	0.022
Lesion with worst Post-PCI TIMI flow, n (%)					0.646					0.002
0 or 1	14 (0.6%)	8 (0.5%)	5 (1.0%)	1 (1.2%)	0.351	63 (1.1%)	42 (0.9%)	15 (1.8%)	6 (2.9%)	0.004
2	67 (3.0%)	51 (3.0%)	13 (2.6%)	3 (3.5%)	0.814	198 (3.5%)	149 (3.2%)	38 (4.6%)	11 (5.3%)	0.055
3	2188 (96.4%)	1618 (96.5%)	489 (96.4%)	81 (95.3%)	0.847	5364 (95.4%)	4400 (95.8%)	774 (93.6%)	190 (91.8%)	0.001
Complete revascularization, n (%)					0.001					<0.001
No	558 (24.6%)	445 (26.5%)	99 (19.5%)	14 (16.5%)	0.001	1627 (28.8%)	1391 (30.2%)	210 (25.3%)	26 (12.6%)	<0.001
Yes	1712 (75.4%)	1232 (73.5%)	409 (80.5%)	71 (83.5%)	0.001	4016 (71.2%)	3215 (69.8%)	620 (74.7%)	181 (87.4%)	<0.001

CABG, coronary artery bypass graft; COPD, chronic obstructive pulmonary disease; CVE, cerebrovascular event; eGFR, estimated granular filtration ratio; GDMT, guideline-directed medical therapy; PCI, percutaneous coronary intervention; STEMI, ST-segment elevation myocardial infarction; TIMI, Thrombolysis In Myocardial Infarction.

Depicted are counts (%) or means ± standard deviations.

PCI characteristics are shown in Table 1. Left anterior descending artery lesions were more frequently observed in patients with GDMT class 4, whereas patients with lower GDMT class had a higher prevalence of right coronary artery lesions. The majority of patients had single-vessel disease, and there was no statistically significant difference in the prevalence of multivessel disease (≥3 vessels) across GDMT classes. At baseline, Thrombolysis In Myocardial Infarction (TIMI) flow grade 0 or 1 was observed in 68.4% (1547/2261) of patients in the pooled randomised cohort and in 72.5% (4073/5621) of patients in the real-world PCI registry. In both cohorts, TIMI flow grade 3 was achieved in the majority of patients (96.4% [2188/2269] and 95.4% [5364/5625], respectively); however, there was a statistically significant difference in the rate of post-procedural TIMI flow grade 3 across GDMT classes in the real-world PCI registry. Complete revascularisation, defined as revascularization of all lesions within the infarct-related vessel, was more frequently achieved at the index PCI in patients with lower GDMT class, while staged PCI was more frequently performed in patients with higher GDMT class.

### Clinical outcomes according to GDMT class

Clinical outcomes according to GDMT class at discharge in the pooled randomised cohort are summarised in Figs. 3 and 4, Table 2 and Supplementary Table S5. The incidence of the composite of cardiovascular death, stroke, or myocardial infarction was higher in patients with GDMT class 0–2 compared with those with GDMT class 3 or 4 throughout follow-up. In multivariable analysis, GDMT class 4 and 3 were associated with a lower risk of the composite outcome at 5 years compared with GDMT class 0–2 (adjusted HR [95% CI]: 0.49 [0.33–0.72] and 0.49 [0.28–0.85], respectively), whereas no statistically significant difference was observed between GDMT class 4 and 3 at discharge (adjusted HR [95% CI]: 0.99 [0.85–1.16]). In the additional analysis of specific pharmacological combinations comprising GDMT class 3, associations with the composite outcome varied across individual class 3 combinations and follow-up periods compared with GDMT class 4 (Supplementary Table S6).

**Fig. 3:**
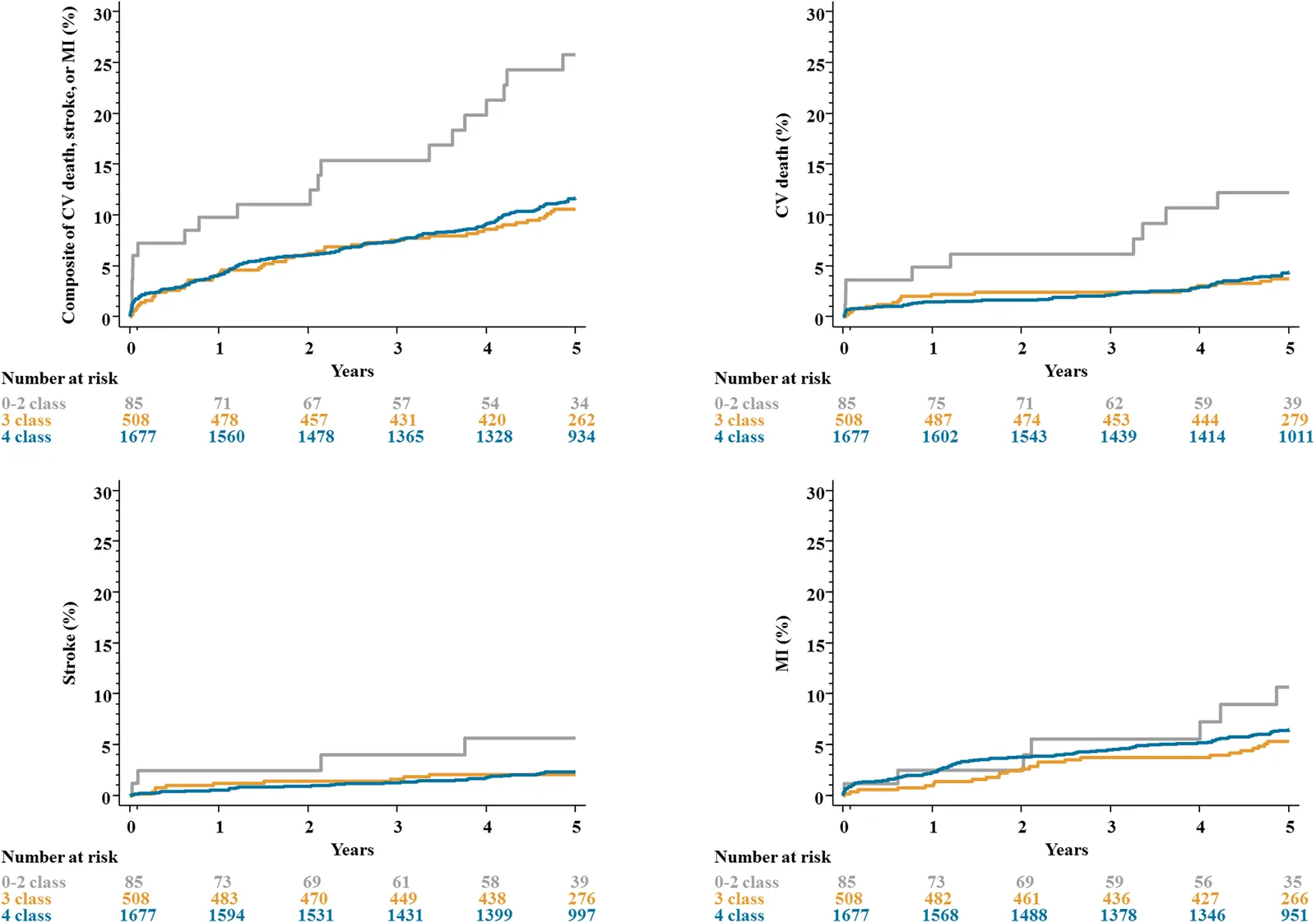
**Clinical outcomes stratified by GDMT class at discharge after STEMI in the pooled randomised cohort.** Time-to-event curves show the cumulative incidence of clinical outcomes stratified by GDMT class stratified at discharge in the pooled randomised cohort. CV, cardiovascular; MI, myocardial infarction. Other abbreviations as in Fig. 1.

**Fig. 4:**
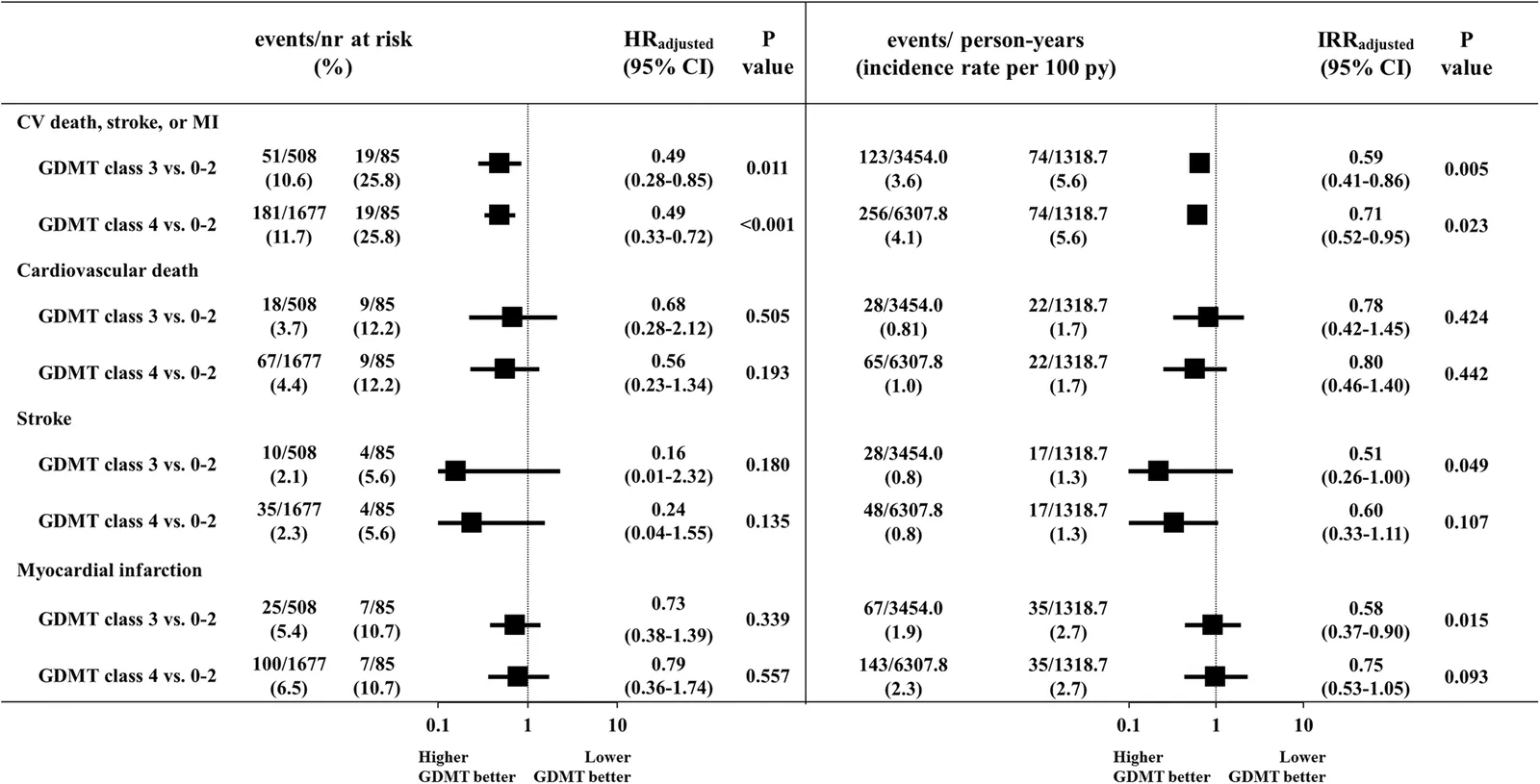
**Association between GDMT class and clinical outcomes after STEMI. Forest plots** show adjusted hazard ratios (HRs) with 95% confidence intervals (CIs) for clinical outcomes according to GDMT class at discharge (**left**) and adjusted incidence rate ratios (IRRs) with 95% CIs from the time-dependent analysis (**right**). Abbreviations as in Fig. 1, Fig. 2, Fig. 3.

**Table 2:** Clinical outcomes stratified by GDMT class at discharge in the pooled randomised cohort.

	GDMT 4 class at discharge N = 1677	GDMT 3 class at discharge N = 508	GDMT 0–2 class at discharge N = 85	GDMT class 4 versus 0–2		GDMT class 3 versus 0–2		GDMT class 4 versus 3	
				Adjusted HR (95% CI)	Adjusted P value	Adjusted HR (95% CI)	Adjusted P value	Adjusted HR (95% CI)	Adjusted P value
At 30 days									
Composite of CV death, stroke, or myocardial infarction, n (%)	29 (1.7%)	5 (1.0%)	5 (6.0%)	0.38 (0.06–2.40)	0.305	0.31 (0.09–1.02)	0.054	1.24 (0.65–2.35)	0.508
Cardiovascular death, n (%)	13 (0.8%)	2 (0.4%)	3 (3.6%)	0.62 (0.29–1.33)	0.220	0.30 (0.01–6.52)	0.446	2.05 (0.04–93.83)	0.713
Myocardial infarction, (%)	15 (0.9%)	2 (0.4%)	1 (1.2%)	0.49 (0.46–0.52)	<0.001	0.40 (0.21–0.77)	0.006	1.22 (0.59–2.49)	0.593
Stroke, n (%)	3 (0.2%)	1 (0.2%)	1 (1.2%)	0.16 (0.00–25.37)	0.477	0.18 (0.00–17.39)	0.460	0.89 (0.54–1.46)	0.646
All-cause death, n (%)	14 (0.8%)	3 (0.6%)	3 (3.6%)	0.63 (0.33–1.21)	0.168	0.60 (0.18–1.92)	0.386	1.07 (0.17–6.57)	0.946
At 1 year									
Composite of CV death, stroke, or myocardial infarction, n (%)	68 (4.1%)	21 (4.2%)	8 (9.8%)	0.47 (0.36–0.60)	<0.001	0.58 (0.21–1.62)	0.300	0.80 (0.37–1.72)	0.566
Cardiovascular death, n (%)	24 (1.5%)	11 (2.2%)	4 (4.9%)	1.41 (0.95–2.09)	0.086	2.72 (1.34–5.51)	0.006	0.52 (0.17–1.56)	0.243
Myocardial infarction, (%)	38 (2.3%)	5 (1.0%)	2 (2.5%)	0.63 (0.44–0.90)	0.011	0.47 (0.40–0.55)	<0.001	1.35 (0.80–2.27)	0.267
Stroke, n (%)	9 (0.6%)	6 (1.2%)	2 (2.5%)	0.13 (0.02–0.63)	0.012	0.20 (0.01–2.85)	0.236	0.62 (0.22–1.72)	0.358
All-cause death, n (%)	34 (2.1%)	13 (2.6%)	5 (6.1%)	0.99 (0.54–1.80)	0.965	1.52 (1.06–2.17)	0.022	0.65 (0.25–1.70)	0.379
At 5 years									
Composite of CV death, stroke, or myocardial infarction, n (%)	181 (11.7%)	51 (10.6%)	19 (25.8%)	0.49 (0.33–0.72)	<0.001	0.49 (0.28–0.85)	0.011	0.99 (0.85–1.16)	0.929
Cardiovascular death, n (%)	67 (4.4%)	18 (3.7%)	9 (12.2%)	0.56 (0.23–1.34)	0.193	0.68 (0.22–2.12)	0.505	0.82 (0.63–1.06)	0.137
Myocardial infarction, (%)	100 (6.5%)	25 (5.4%)	7 (10.7%)	0.79 (0.36–1.74)	0.557	0.73 (0.38–1.39)	0.339	1.08 (0.94–1.25)	0.270
Stroke, n (%)	35 (2.2%)	10 (2.1%)	4 (5.6%)	0.24 (0.04–1.55)	0.135	0.16 (0.01–2.32)	0.180	1.49 (0.67–3.30)	0.328
All-cause death, n (%)	113 (7.3%)	32 (6.6%)	12 (15.9%)	0.80 (0.46–1.37)	0.407	0.86 (0.37–2.02)	0.733	0.92 (0.68–1.26)	0.617

CV = cardiovascular; GDMT = guideline-directed medical therapy; MI = myocardial infarction.

Kaplan–Meier estimates were used to calculate cumulative event rates, and Cox proportional hazards models were used to estimate hazard ratios (HRs) with 95% confidence intervals (CIs). Models were adjusted for clustering by trial. Only the first occurrence of each event type was considered for each patient.

In the real-world PCI registry, a higher incidence of the composite outcome at 30 days and 1 year was observed in patients with GDMT class 0–2, as compared with those with GDMT class 4 and 3. Consistent with the pooled randomised cohort, GDMT class 4 and 3 at discharge were associated with a lower risk of the composite outcome compared with GDMT class 0–2. In contrast to the pooled randomised cohort, GDMT class 4 at discharge was associated with a lower, albeit statistically borderline significant, risk of the composite outcome compared with GDMT class 3 (adjusted HR [95% CI]: 0.73 [0.54–1.00]) (Supplementary Tables S7 and S8). GDMT class 4 was also associated with a lower risk of all-cause and cardiovascular death at 30 days and 1 year compared with GDMT class 3 and 0–2, whereas there were no statistically significant differences in the risk of stroke or myocardial infarction across GDMT classes (Supplementary Figure S2). The proportional hazards assumption was violated for the composite cardiovascular outcome, cardiovascular death, stroke, and all-cause death in the real-world PCI registry. In additional piecewise Cox analyses with follow-up divided at 180 days, differences in the composite cardiovascular outcome and mortality across GDMT classes were predominantly observed during the first 180 days, whereas no statistically significant differences were observed between 181 and 365 days (Supplementary Table S9).

### Subgroup analysis

The results of the subgroup analyses across age, sex, Killip class, left ventricular ejection fraction, and major comorbidities, are presented in Fig. 5. Point estimates generally favoured GDMT class 3–4 over GDMT class 0–2 across the examined subgroups, with no statistically significant interaction.

**Fig. 5:**
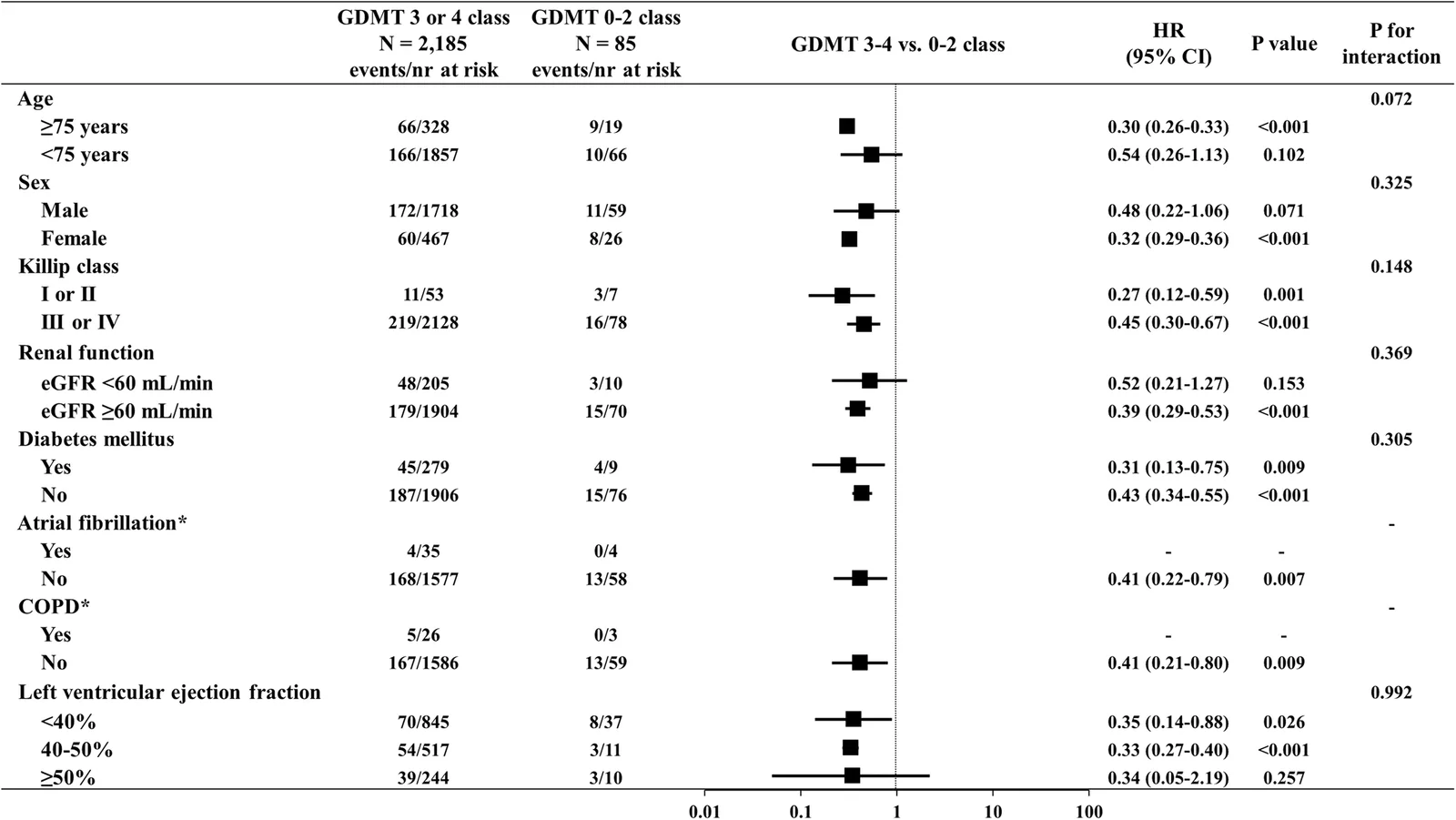
S**tratified analyses of composite of cardiovascular death, stroke, or myocardial infarction at 5 years after STEMI across subgroups in the pooled randomised cohort.** Time-to-event curves show the cumulative incidence of clinical outcomes stratified by GDMT class at discharge in the real-world PCI registry. Cox proportional hazards models were used to estimate hazard ratios (HRs) with 95% confidence intervals (95% CIs) for the composite outcome within each prespecified subgroup. P values were derived from Wald chi-square tests. Interaction P values were obtained from likelihood ratio tests comparing models with and without interaction terms. Models were adjusted for clustering by trial. ∗not captured in COMFORTABLE-AMI. COPD, chronic obstructive pulmonary disease; eGFR, estimated glomerular filtration rate. Other abbreviations as in Fig. 1, Fig. 2, Fig. 3, Fig. 4.

### Sensitivity analysis

In the sensitivity analysis, the direction of the associations was generally consistent with the main analysis (Supplementary Tables S10–S13).

### Time-dependent analysis

In the time-dependent analysis, a total of 256 composite outcome events occurred over 6307.8 person-years in patients with GDMT class 4 (incidence rate per 100 person-years [95% CI]: 4.06 [3.58–4.59]), 123 events over 3454.0 person-years in patients with GDMT class 3 (3.56 [2.96–4.25]), and 74 events over 1318.7 person-years in patients with GDMT class 0–2 (5.61 [4.41–7.04]), respectively (Fig. 4 and Table 3). GDMT class 4 and 3 were associated with a lower risk of the composite outcome compared with GDMT class 0–2 (adjusted IRR [95% CI]: 0.71 [0.52–0.95] and 0.59 [0.41–0.86], respectively), while no statistically significant difference was observed between GDMT class 4 and 3 (adjusted IRR [95% CI]: 1.19 [0.94–1.50]). All results of the time-dependent analysis are described in Fig. 4, Table 3, and Supplementary Table S14.

**Table 3:** Analysis of clinical outcomes according to time-dependent GDMT class in the pooled randomised cohort.

	GDMT 4 class		GDMT 3 class		GDMT 0–2 class		GDMT class 4 versus 0–2		GDMT class 3 versus 0–2		GDMT class 4 versus 3	
	Events/person-years	Incidence rate per 100 person-years (95% CI)	Events/person-ears	Incidence rate per 100 person-years (95% CI)	Events/person-ears	Incidence rate per 100 person-years (95% CI)	Adjusted IRR (95% CI)	Adjusted P value	Adjusted IRR (95% CI)	Adjusted P value	Adjusted IRR (95% CI)	Adjusted P value
Composite of CV death, stroke, or myocardial infarction	256/6307.8	4.06 (3.58–4.59)	123/3454.0	3.56 (2.96–4.25)	74/1318.7	5.61 (4.41–7.04)	0.71 (0.52–0.95)	0.023	0.59 (0.41–0.86)	0.005	1.19 (0.94–1.50)	0.151
Cardiovascular death	65/6307.8	1.03 (0.80–1.31)	28/3454.0	0.81 (0.54–1.17)	22/1318.7	1.67 (1.05–2.53)	0.80 (0.46–1.40)	0.442	0.78 (0.42–1.45)	0.424	1.04 (0.64–1.69)	0.890
Myocardial infarction	143/6307.8	2.27 (1.91–2.67)	67/3454.0	1.94 (1.50–2.46)	35/1318.7	2.65 (1.85–3.69)	0.75 (0.53–1.05)	0.093	0.58 (0.37–0.90)	0.015	1.30 (0.96–1.75)	0.085
Stroke	48/6307.8	0.76 (0.56–1.01)	28/3454.0	0.81 (0.54–1.17)	17/1318.7	1.29 (0.75–2.06)	0.60 (0.33–1.11)	0.107	0.51 (0.26–1.00)	0.049	1.20 (0.77–1.85)	0.422
All-cause death	96/6307.8	1.52 (1.23–1.86)	42/3454.0	1.22 (0.88–1.64)	37/1318.7	2.81 (1.98–3.87)	0.73 (0.46–1.17)	0.193	0.62 (0.36–1.07)	0.086	1.18 (0.77–1.80)	0.448

CV = cardiovascular; GDMT = guideline-directed medical therapy; MI = myocardial infarction.

Only post-discharge outcomes were included in this analysis. GDMT class was treated as a time-dependent variable and updated at 1 and 2 years if different from the previous period. Incidence rates are expressed per 100 person-years. Poisson regression models with log(person-time) as an offset were used to estimate incidence rate ratios (IRRs) with 95% confidence intervals (CIs). Mixed-effects Poisson regression models with a random effect for patient ID were used to account for within-patient correlation. All event occurrences during each GDMT class period were included in the analysis.

## Discussion

In the present pooled observational analysis of three investigator-initiated randomised trials, with external validation in a real-world PCI registry, we evaluated the implementation and long-term persistence of four-class GDMT after STEMI across successive treatment eras. The principal findings of this study can be summarised as follows. First, more than two-thirds of patients received GDMT class 4 at discharge after STEMI, whereas fewer than 5% were prescribed GDMT class ≤2 across study cohorts and periods; however, the proportion of patients receiving GDMT class 4 gradually declined during follow-up, with a corresponding increase in the proportion of patients with GDMT class 0–2. Second, GDMT class was associated with patient baseline profile; patients who received lower GDMT class after STEMI were more likely to be female, had a higher prevalence of Killip class III or IV and a greater comorbidity burden, with these differences more pronounced in the real-world PCI registry. Third, in exploratory outcome analyses, higher GDMT class at discharge and during follow-up was associated with a lower risk of the composite cardiovascular outcome; these associations should be interpreted in the context of substantial differences in baseline clinical risk across GDMT classes.

Despite substantial improvements in quality of STEMI care, long-term outcomes remain suboptimal. In an analysis from the Western Denmark Heart Registry including 18,818 patients with first-time STEMI undergoing primary PCI between 2003 and 2018 (median age; 64 years, 73.5% male), patients who survived the initial 90 days continued to have higher long-term mortality than age- and sex-matched individuals from the general population. Of note, the excess mortality was more apparent beyond 6 years of follow-up, and this pattern was observed in both earlier (2003–2010) and more contemporary (2011–2018) treatment eras.^4^ Similarly, the nationwide SWEDEHEART registry showed that although the loss in life expectancy after myocardial infarction has decreased over time, improvements have plateaued in recent years, with excess risk particularly among younger patients and those with impaired left ventricular function.^18^ Indeed, despite advances in DES technologies and pharmacological therapies across successive treatment eras represented by the randomised trials included in the present study, the 5-year rates of major ischaemic outcomes appeared broadly comparable across trials.^11^, ^15^ These findings highlight the substantial residual risk after STEMI, underscoring the importance of effective and sustained secondary prevention strategies.

It is well-known that the implementation and persistence of GDMT in the context of secondary prevention of cardiovascular disease remain challenging.^19^, ^20^ In an analysis using a national insurance claim database in South Korea (2013–2017), the prescription rate of optimal medical therapy, defined as a combination of antiplatelet agents, statins, renin-angiotensin system inhibitors, and beta-blockers, was 57.1% at discharge after acute myocardial infarction and continued to decrease to 20.9% at 3 years.^8^ In the present study, approximately 70–90% of patients with STEMI received GDMT class 4 at discharge. This relatively high prescription rate, observed in both the randomised trials and the real-world PCI registry, may reflect the inclusion of a more homogeneous population of patients with STEMI with high-risk clinical entity compared with broader populations of acute myocardial infarction. Nevertheless, in the present study, the high prescription rates of GDMT class 4 were not sustained during follow-up in both the randomised trials and the real-world PCI registry. Incomplete implementation and declining persistence of secondary prevention therapies in cardiovascular medicine are likely multifactorial, including physician-, patient-, therapy-, and healthcare system-related factors.^21^ Furthermore, patient adherence plays a critical role in long-term secondary prevention. In an earlier analysis using the data of U.S Medicare Database (2008–2010), only 49% of patients after acute myocardial infarction adhered to all statins, renin-angiotensin system inhibitors, and beta-blockers within the first 180 days after the index hospitalisation.^22^

In this study, patients with a higher GDMT class at discharge, as well as those with a higher GDMT class during follow-up, had a lower risk of the composite cardiovascular outcome compared with those with a lower GDMT class. However, these exploratory associations require cautious interpretation in light of marked differences in clinical profile across GDMT classes. Whilst more than 90% of patients achieved a final TIMI flow grade of 3 at the time of primary PCI, patients with lower GDMT class had more severe clinical presentations, including a higher prevalence of Killip class III-IV, and a greater burden of comorbidities. Previous studies have consistently shown that patients with higher clinical risk are less likely to receive optimal secondary prevention and are at increased risk of adverse clinical outcomes.^9^, ^20^, ^23^ Lower GDMT class may reflect greater disease severity, haemodynamic instability, contraindications, or other clinical circumstances limiting the initiation or continuation of individual therapies, rather than undertreatment alone. Residual confounding and confounding by indication cannot be excluded despite multivariable adjustment, and these observational analyses cannot establish whether initiation or continuation of additional GDMT classes would have reduced subsequent event rates. These considerations may also partly explain the observed differences in clinical outcomes between the pooled randomised cohort and the real-world PCI registry. Within the real-world PCI registry, differences in outcomes across GDMT classes were pronounced early after discharge, particularly for mortality. This temporal pattern may partly reflect the greater underlying clinical severity of patients receiving fewer GDMT classes. Similarly, although the time-dependent analysis accounted for changes in GDMT status over time, treatment modifications may have been influenced by evolving clinical status, as well as early clinical events, which could not be fully captured in the present analysis. Moreover, classification according to the number of prescribed drug classes grouped patients receiving different pharmacological combinations within the same GDMT class. In an exploratory analysis of specific GDMT class 3 combinations, associations with cardiovascular outcomes varied across individual treatment combinations, highlighting the heterogeneity inherent in a classification based solely on the number of prescribed drug classes. However, given the small numbers of patients and events for several combinations and the same potential for confounding by indication, these analyses do not permit conclusions regarding the relative efficacy or dispensability of individual GDMT components.

The present findings highlight two considerations for contemporary secondary prevention after STEMI: the challenge of maintaining established pharmacological therapies over long-term follow-up and the need to place these therapies within an evolving and increasingly individualised framework of secondary prevention. The use of quality indicators, community-based follow-up, and improved digital health infrastructure may facilitate better implementation, persistence, and adherence to GDMT.^9^, ^24^ The four pharmacological classes evaluated in the present study represent established components of secondary prevention but do not encompass the full spectrum of contemporary care, which also includes lifestyle modification, cardiac rehabilitation, intensive risk-factor management, and newer pharmacological therapies in appropriately selected patients. The present study did not evaluate the implementation or clinical impact of these additional components of contemporary secondary prevention. Novel pharmacological approaches and updated treatment strategies have emerged. For example, recent heart failure guidelines advocate the early and comprehensive initiation of foundational therapies (renin-angiotensin system inhibitors, beta-blockers, mineralocorticoid receptor antagonists, and sodium-glucose co-transporter 2 inhibitors), which may inform GDMT strategies after STEMI.^25^ However, the incremental clinical benefit of novel pharmacological therapies, including mineralocorticoid receptor antagonists and sodium-glucose co-transporter 2 inhibitors, beyond established standard of care has not been clearly established in patients with STEMI.^26^, ^27^, ^28^ Conversely, emerging evidence has also prompted re-evaluation of the routine initiation or long-term continuation of individual GDMT components in selected patients. Specifically, recent studies have re-evaluated the role of beta-blocker therapy in patients with acute myocardial infarction and preserved left ventricular systolic function.^29^ The optimal composition and duration of GDMT after STEMI may require further refinement, and treatment decisions should continue to be individualised according to patient risk profile and evolving evidence. Against this evolving therapeutic landscape, our findings highlight persistent challenges in the long-term implementation and persistence of established pharmacological therapies after STEMI, particularly among patients with greater clinical complexity. Recent global initiatives have emphasised the importance of prevention and early detection of coronary atherosclerosis and high-risk individuals, rather than focussing solely on the treatment of established myocardial ischaemia.^30^ A simulation study suggested that up to 82.1% of atherosclerotic cardiovascular disease deaths could be prevented by 2050 if behavioural and metabolic risk factors were controlled.^30^ Although a comprehensive approach is required, such strategies have the potential to halt, delay, or even reverse the progression of disease, reducing the long-term burden of cardiovascular events.

Results of the present study should be interpreted in light of several limitations. First, this study was observational in nature and hypothesis-generating, and GDMT prescription was not randomised. Residual confounding and confounding by indication cannot be excluded despite multivariable adjustment, and the observed associations between GDMT class and clinical outcomes should not be interpreted as causal. Second, the four-class GDMT framework used in the present study was selected to enable consistent assessment across the treatment eras represented by the study cohorts and should not be interpreted as encompassing all components of contemporary secondary prevention or optimal medical therapy after STEMI. Detailed information on medication dose and intensity, as well as reasons for non-initiation, modification, or discontinuation, was not available. In addition, prescription data do not necessarily reflect actual patient adherence. Third, although definitions of STEMI and clinical endpoints were broadly consistent across study cohorts, differences in the specific definitions and inclusion and exclusion criteria may have influenced the comparability of results. Furthermore, temporal changes in clinical practice, including advances in PCI techniques, pharmacological therapies, and the availability of newer medical agents over the study period, may have affected both treatment patterns and outcomes. Follow-up of BIOSTEMI and the real-world PCI registry also partly overlapped with the COVID-19 pandemic, which may have influenced healthcare delivery, treatment persistence, and clinical outcomes in ways that could not be fully accounted for in the present analyses. Moreover, external validation was performed using data from a single-centre real-world PCI registry in Switzerland with relatively short follow-up duration. Differences in healthcare organisation, follow-up practices, and prescribing patterns across healthcare settings may limit the generalisability of these findings. Race and ethnicity data were not systematically collected in the source studies and were not available for the present analysis. Nevertheless, the registry provided a contemporary real-world STEMI cohort with prospective data collection and largely consistent definitions of STEMI and clinical endpoints, enabling assessment of the consistency of the observed longitudinal patterns in routine clinical practice. Finally, although the analyses accounted for clustering, residual heterogeneity across the three randomised trials may not have been fully captured.

### Conclusion

More than two-thirds of patients received GDMT class 4 at discharge after STEMI, while the proportion declined during follow-up, with a corresponding increase in patients receiving GDMT class 0–2. In exploratory analyses, higher GDMT class was associated with a lower risk of cardiovascular events. These findings highlight challenges in the long-term implementation and persistence of established pharmacological secondary prevention after STEMI, while underscoring the need for individualised treatment within an evolving contemporary secondary prevention framework.

## Contributors

Conceptualisation (DT, TP), Data curation (DH, SL), Formal analysis (DH), Funding acquisition (LR, TP), Investigation (DH, SL, MR, JDH, LH, MF, NC, JL, FP, OM, JFI, SW, LR, TP), Methodology (DT, DH, TP), Project administration (DT, TP), Resources (DH, SL, LR, TP), Supervision (SW, LR, TP), Validation (DH, TP), Visualisation (DT), Writing—original draft (DT, TP), and Writing—review & editing (All authors). The data underlying this article were provided by the CTU, University of Bern. TP and DH had full access to all the data in the study and take responsibility for the integrity of the data and the accuracy of the data analysis. The authors were not precluded from accessing the data, and the corresponding author had final responsibility for the decision to submit for publication.

## Data sharing statement

The data that support the findings of this study are not publicly available. Requests for access to the data may be submitted to the corresponding author and will be considered in accordance with applicable ethical, legal, and institutional requirements.

## Declaration of interests

Dr. Pilgrim reports research, travel or educational grants to the institution without personal remuneration from Biotronik, Boston Scientific, Edwards Lifesciences, Medtronic, Abbott, and ATSens; speaker fees and consultancy fees to the institution from Biotronik, Boston Scientific, Edwards Lifesciences, Abbott, Medtronic, Biosensors, and Highlife; and receipt of equipment/materials/services from ATsens. Dr Pilgrim served on a Biosensors Data Safety Monitoring Board/Advisory Board relationship, as well as on a HighLife Clinical Event Adjudication Committee. Dr. Räber received speaker/consultation fees by Abbott, Boston Scientific, Novo Nordisc, Occlutech and Sanofi, and research grants to the institution by Abbott, Boston Scientific, Heartflow, Novo Nordic, Swiss National Science Foundation, Swiss Heart Foundation. Dr. Windecker reports research, travel or educational grants to the institution without personal remuneration from Abbott, Abiomed, Amgen, Anteris, Alnylam, Amicus Therapeutics, Astra Zeneca, Bayer, Braun, Bioanalytica, Biotronik, Boehringer Ingelheim, Boston Scientific, Bristol Myers Squibb, Cordis Medical, Corflow Therapeutics, CSL Behring, Daiichi Sankyo, Edwards Lifesciences, Eli Lilly (Suisse), EnCarda Inc, Fumedica, GE Healthcare, Guerbet, IACULIS, Inari Medical, Janssen AI, Johnson & Johnson, Linde Gas, M.A. Medalliance, Medtronic, MSD Merck, Sharp & Dohme, Novartis, Novo Nordisk, OM Pharma, Orchestra BioMed, Pfizer, Philips AG, Sanofi-Aventis, Servier, Siemens Healthcare, Sinomed, SMT Sahajanand Medical Technologies, TRiCares GmbH, Vascular Medical, V-Wave, and ZOLL Medical. Dr. Windecker served as advisory board member and/or member of the steering/executive group of trials funded by Abbott, Abiomed, Amgen, Anteris, Astra Zeneca, Bayer, Boston Scientific, Biotronik, Bristol Myers Squibb, Edwards Lifesciences, Medtronic, Novartis, Sinomed, and V-Wave with payments to the institution but no personal payments. He is also an Expert Panel Member of the Clinical Study Group of the Deutsches Zentrum für Herz-Kreislauf Forschung and the Global Cardiovascular Research Funder Forum, Secretary/Treasurer of the ESC Board and Management Group, Associate Editor of JAMA Cardiology, Section Editor of UpToDate, and a member of the Board of Directors of the Swiss Heart Foundation with no personal payments. Dr. Iglesias reports unrestricted research grants to the institution from Biotronik, CathWorks, Concept Medical, SMT, Terumo Corp, and the De Reuter Foundation outside of the submitted work; consultant fees from Cordis; honoraria/speaker's fee to the institution: Cordis, Concept Medical, Medtronic, Penumbra Inc., Philips Volcano, Recor Medical, and Teleflex; and travel fees from Biotronik, Cordis and Medtronic. Dr. Muller reports educational grants to the institution without personal remuneration from Edwards Lifesciences and Abbott; honoraria/speaker's fee to the institution from Edwards Lifesciences, Abbott; and support for attending meetings and/or travel from Abbott and Edwards Lifesciences. Dr. Praz reports research grants to the institution from Abbott Vascular and Edwards Lifesciences; travel expenses from Abbott Vascular, Edwards Lifesciences, Medira, Siemens Healthineers, and InQB8 Medical Technologies; and honoraria/speaker's fee to the institution from Abbott Vascular and Edwards Lifesciences. Dr. Lanz reports a dedicated research grant from Edwards Lifesciences; travel expenses from Abbott Vascular and Edwards Lifesciences. Dr. Rohla reports advisory fees from Daiichi Sankyo, Sanofi Aventis, COR2ED, Novartis, Medtronic and OM Pharma and lecturing fees from Daiichi Sankyo, Biotronik, Medtronic and Takeda Pharma. Drs. Heg and Losdat are affiliated with the Department of Clinical Research (DCR) of the University of Bern, which follows a staff policy of not accepting honoraria or consultancy fees. However, the DCR is involved in the design, conduct, or analysis of clinical studies funded by not-for-profit and for-profit organisations. In particular, pharmaceutical and medical device companies provide direct funding to some of these studies. For an up-to-date list of the DCR's funding sources see https://dcr.unibe.ch/services/declaration_of_interest/index_eng.html. All other authors have no relationships to disclose that are relevant to the contents of this article.

## Figures and Tables

**eFigure 1:**
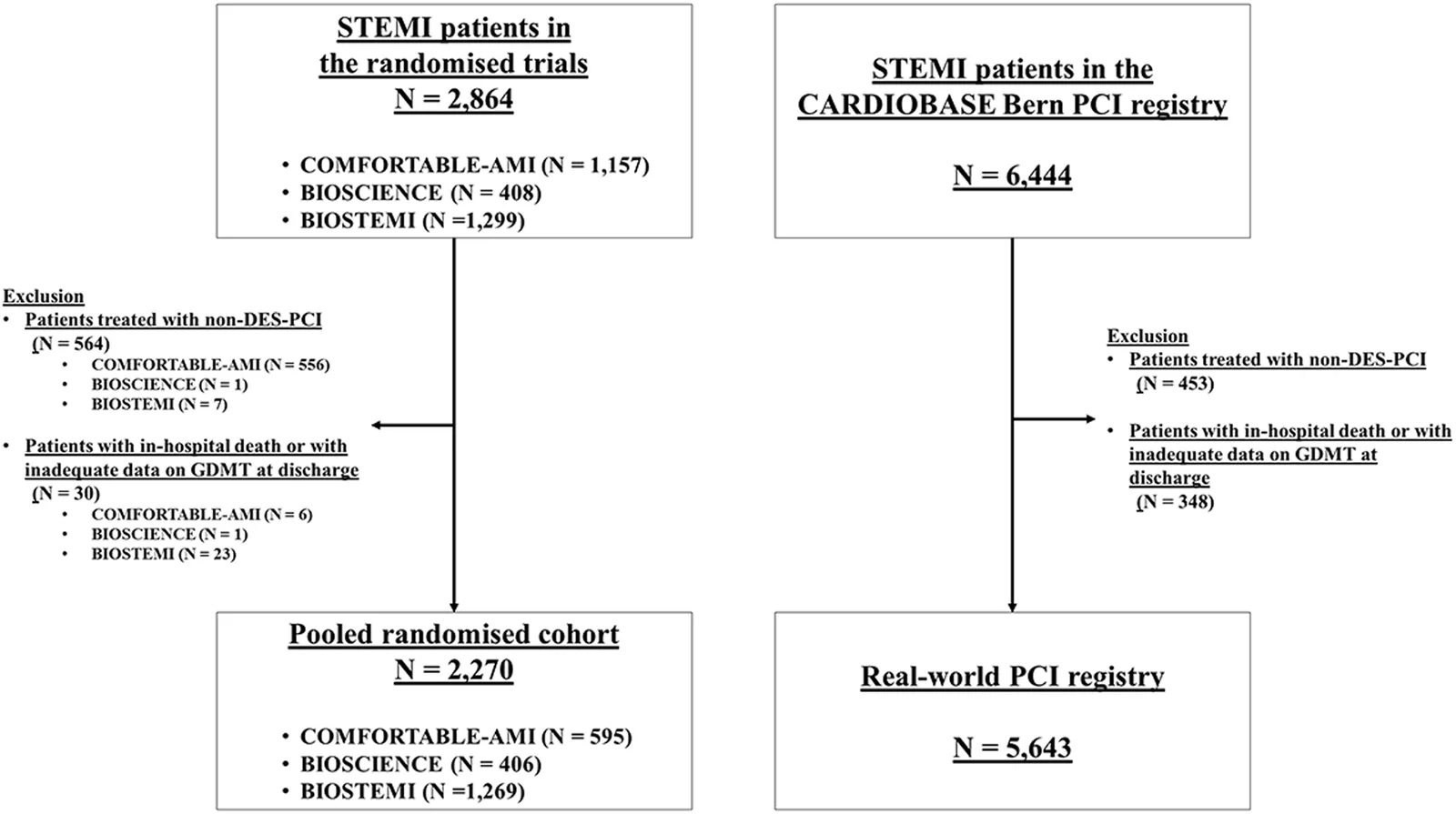


**eFigure 2:**
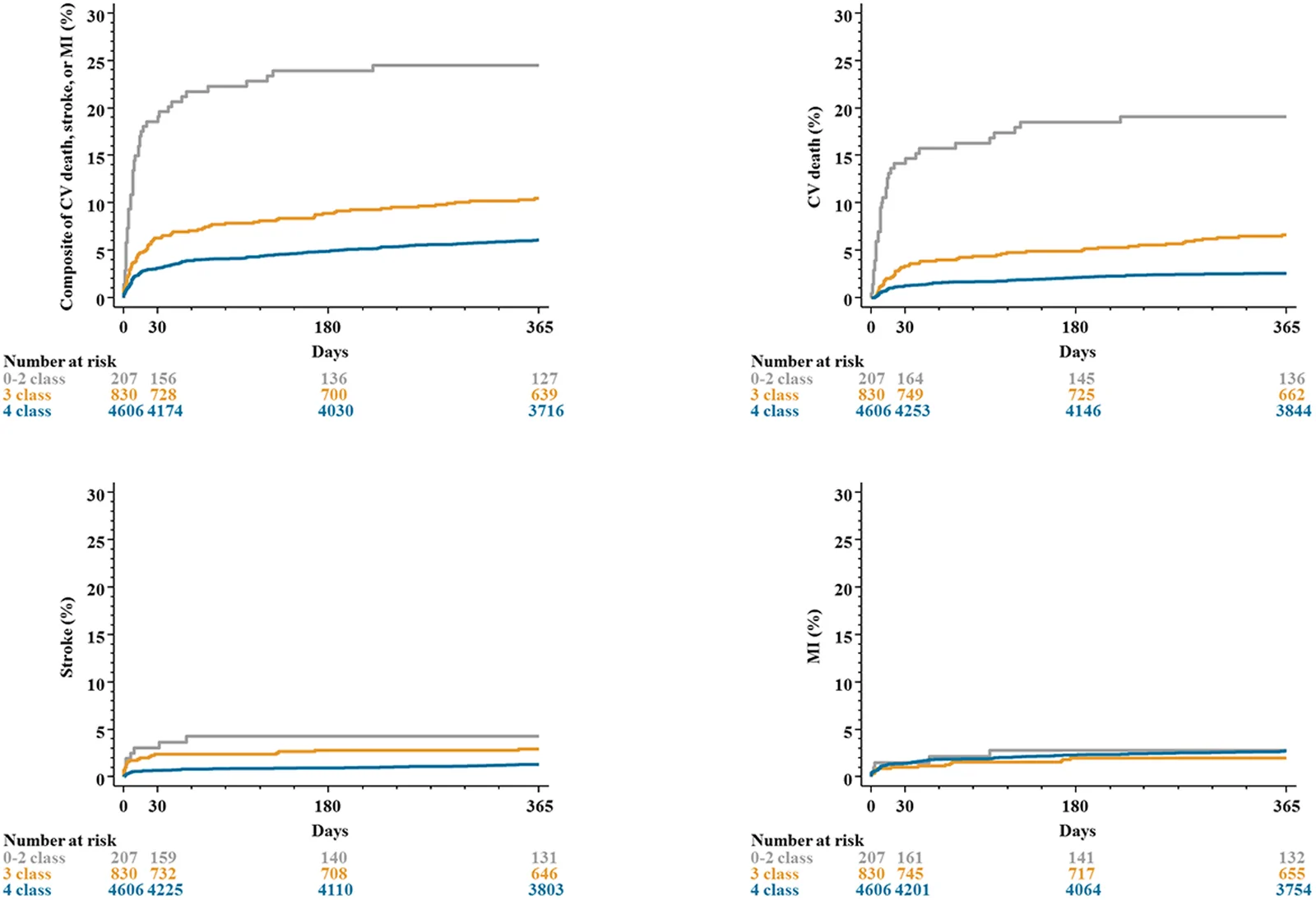


## Appendix A Supplementary data

Cover Letter

Revised Supplemental Materials
