# Supplementary material for: Guideline-directed medical therapy use, persistence, and cardiovascular outcomes following ST-segment elevation myocardial infarction: a pooled analysis of three randomised trials with external registry validation
Source: Lancet Reg Health Eur. 2026 Nov 7;72:101919. doi: 10.1016/j.lanepe.2026.101919 (PMC13763075; doi:10.1016/j.lanepe.2026.101919)
Supplement: Supplementary file 1 [file mmc1.docx]

Bern, October 20th 2026

**TLRHEUROPE-D-26-01096R2**

**Guideline-directed medical therapy use, persistence, and cardiovascular outcomes following ST-segment elevation myocardial infarction: a pooled analysis of three randomised trials with external registry validation**

Dear Prof. Jha,

Dear Prof. Oterio,

We thank the Editors for the careful review of our revised manuscript and appreciate the opportunity to submit a newly revised version of the above-mentioned manuscript.

The manuscript has been revised according to the comments of the Editors. Each point raised by the Editors has been carefully addressed and laid out in a separate response letter.

We believe that the revised version of the manuscript has been further improved and hope to have satisfactorily addressed all of the raised issues in the revised version of this manuscript.

All authors have participated in the work and have reviewed and agree with the content of the article.

Again, we thank you for evaluating the revised manuscript and hope that you will find it suitable for publication in *THE LANCET Regional Health - Europe*.

Sincerely,

Thomas Pilgrim, MD, MSc

**Sources of Funding:** The COMFORTABLE AMI trial was supported by an unrestricted grant from Biosensors SA, Morges, Switzerland. The BIOSCIENCE trial was supported by an unrestricted grant from Biotronik, Bülach, Switzerland. The BIOSTEMI trial was supported by a dedicated research grant from Biotronik, Bülach, Switzerland. The CARDIOBASE Bern PCI registry is supported by institutional funding and infrastructure from Insel Gruppe AG, University Hospital Bern, Bern, Switzerland.

**Role of Funder:** The funders had no role in the design and conduct of the study; collection, management, analysis, and interpretation of the data; preparation, review, or approval of the manuscript; and the decision to submit the manuscript for publication.

**Disclosures:** Dr. Pilgrim reports research, travel or educational grants to the institution without personal remuneration from Biotronik, Boston Scientific, Edwards Lifesciences, Medtronic, Abbott, and ATSens; speaker fees and consultancy fees to the institution from Biotronik, Boston Scientific, Edwards Lifesciences, Abbott, Medtronic, Biosensors, and Highlife; and receipt of equipment/materials/services from ATsens. Dr Pilgrim served on a Biosensors Data Safety Monitoring Board/Advisory Board relationship, as well as on a HighLife Clinical Event Adjudication Committee. Dr. Räber received speaker/consultation fees by Abbott, Boston Scientific, Novo Nordisc, Occlutech and Sanofi, and research grants to the institution by Abbott, Boston Scientific, Heartflow, Novo Nordic, Swiss National Science Foundation, Swiss Heart Foundation. Dr. Windecker reports research, travel or educational grants to the institution without personal remuneration from Abbott, Abiomed, Amgen, Anteris, Alnylam, Amicus Therapeutics, Astra Zeneca, Bayer, Braun, Bioanalytica, Biotronik, Boehringer Ingelheim, Boston Scientific, Bristol Myers Squibb, Cordis Medical, Corflow Therapeutics, CSL Behring, Daiichi Sankyo, Edwards Lifesciences, Eli Lilly (Suisse), EnCarda Inc, Fumedica, GE Healthcare, Guerbet, IACULIS, Inari Medical, Janssen AI, Johnson & Johnson, Linde Gas, M.A. Medalliance, Medtronic, MSD Merck, Sharp & Dohme, Novartis, Novo Nordisk, OM Pharma, Orchestra BioMed, Pfizer, Philips AG, Sanofi-Aventis, Servier, Siemens Healthcare, Sinomed, SMT Sahajanand Medical Technologies, TRiCares GmbH, Vascular Medical, V-Wave, and ZOLL Medical. Dr. Windecker served as advisory board member and/or member of the steering/executive group of trials funded by Abbott, Abiomed, Amgen, Anteris, Astra Zeneca, Bayer, Boston Scientific, Biotronik, Bristol Myers Squibb, Edwards Lifesciences, Medtronic, Novartis, Sinomed, and V-Wave with payments to the institution but no personal payments. He is also an Expert Panel Member of the Clinical Study Group of the Deutsches Zentrum für Herz-Kreislauf Forschung and the Global Cardiovascular Research Funder Forum, Secretary/Treasurer of the ESC Board and Management Group, Associate Editor of JAMA Cardiology, Section Editor of UpToDate, and a member of the Board of Directors of the Swiss Heart Foundation with no personal payments. Dr. Iglesias reports unrestricted research grants to the institution from Biotronik, CathWorks, Concept Medical, SMT, Terumo Corp, and the De Reuter Foundation outside of the submitted work; consultant fees from Cordis; honoraria/speaker’s fee to the institution: Cordis, Concept Medical, Medtronic, Penumbra Inc., Philips Volcano, Recor Medical, and Teleflex; and travel fees from Biotronik, Cordis and Medtronic. Dr. Muller reports educational grants to the institution without personal remuneration from Edwards Lifesciences and Abbott; honoraria/speaker’s fee to the institution from Edwards Lifesciences, Abbott; and support for attending meetings and/or travel from Abbott and Edwards Lifesciences. Dr Praz reports research grants to the institution from Abbott Vascular and Edwards Lifesciences; travel expenses from Abbott Vascular, Edwards Lifesciences, Medira, Siemens Healthineers, and InQB8 Medical Technologies; and honoraria/speaker’s fee to the institution from Abbott Vascular and Edwards Lifesciences. Dr. Lanz reports a dedicated research grant from Edwards Lifesciences; travel expenses from Abbott Vascular and Edwards Lifesciences. Dr. Rohla reports advisory fees from Daiichi Sankyo, Sanofi Aventis, COR2ED, Novartis, Medtronic and OM Pharma and lecturing fees from Daiichi Sankyo, Biotronik, Medtronic and Takeda Pharma. Drs. Heg and Losdat are affiliated with the Department of Clinical Research (DCR) of the University of Bern, which follows a staff policy of not accepting honoraria or consultancy fees. However, the DCR is involved in the design, conduct, or analysis of clinical studies funded by not-for-profit and for-profit organizations. In particular, pharmaceutical and medical device companies provide direct funding to some of these studies. For an up-to-date list of the DCR’s funding sources see https://dcr.unibe.ch/services/declaration_of_interest/index_eng.html. All other authors have no relationships to disclose that are relevant to the contents of this article.
