# Supplementary material for: Guideline-directed medical therapy use, persistence, and cardiovascular outcomes following ST-segment elevation myocardial infarction: a pooled analysis of three randomised trials with external registry validation
Source: Lancet Reg Health Eur. 2026 Nov 7;72:101919. doi: 10.1016/j.lanepe.2026.101919 (PMC13763075; doi:10.1016/j.lanepe.2026.101919)
Supplement: Supplementary file 2 [file mmc2.docx]

**SUPPLEMENTARY MATERIAL**

**Guideline-directed medical therapy use, persistence, and cardiovascular outcomes following ST-segment elevation myocardial infarction: a pooled analysis of three randomised trials with external registry validation**

Tomii et al.

**Corresponding Author:**

Thomas Pilgrim, MD

Department of Cardiology

Bern University Hospital, Inselspital

University of Bern

CH-3010 Bern

Phone: +41 31 632 21 11

Fax: +41 31 632 47 70

Mail: thomas.pilgrim@insel.ch

Table of Contents

[Supplementary Information. Trial-specific information, key inclusion and exclusion criteria, and definitions	3](#_Toc240017369)

[Supplementary Table S1. Prescription of GDMT classes after STEMI among patients with available medication information	9](#_Toc240017370)

[Supplementary Table S2. Longitudinal distribution of GDMT classes after STEMI including death and unavailable medication information	11](#_Toc240017371)

[Supplementary Table S3. Temporal trends in prescription GDMT classes in the real-world PCI registry	13](#_Toc240017372)

[Supplementary Table S4. Prescription of GDMT components after STEMI over time	15](#_Toc240017373)

[Supplementary Table S5. Univariable Cox proportional hazards models for clinical outcomes in the pooled randomised cohort	18](#_Toc240017374)

[Supplementary Table S6. Exploratory analysis of the composite outcome according to specific GDMT class 3 combinations	20](#_Toc240017375)

[Supplementary Table S7. Clinical outcomes stratified by GDMT class at discharge in the real-world PCI registry	25](#_Toc240017376)

[Supplementary Table S8. Univariable Cox proportional hazards models for clinical outcomes in the real-world PCI registry	27](#_Toc240017377)

[Supplementary Table S9. Proportional hazards assessment and piecewise Cox analyses for clinical outcomes in the real-world PCI registry	29](#_Toc240017378)

[Supplementary Table S10. Sensitivity analysis: clinical outcomes stratified by GDMT class at discharge in the pooled randomised cohort (only post-discharge outcomes counted)	30](#_Toc240017379)

[Supplementary Table S11. Univariable Cox proportional hazards models for clinical outcomes in the pooled randomised cohort (post-discharge outcomes only)	33](#_Toc240017380)

[Supplementary Table S12. Sensitivity analysis: clinical outcomes stratified by GDMT class at discharge in the real-world PCI registry (only post-discharge outcomes counted)	35](#_Toc240017381)

[Supplementary Table S13. Univariable Cox proportional hazards models for clinical outcomes in the real-world PCI registry (only post-discharge outcomes counted)	38](#_Toc240017382)

[Supplementary Table S14. Univariable Poisson regression models for time-dependent clinical outcomes in the pooled randomised cohort	40](#_Toc240017383)

[Supplementary Figure S1. Study flowchart	42](#_Toc240017384)

[Supplementary Figure S2. Clinical outcomes stratified by GDMT class at discharge after STEMI in the real-world PCI registry	44](#_Toc240017385)

# **Supplementary Information. Trial-specific information, key inclusion and exclusion criteria, and definitions**

|  | **COMFORTABLE-AMI** | **BIOSCIENCE** | **BIOSTEMI** |
| --- | --- | --- | --- |
| **Enrolment period** | September 2009 – January 2011 | February 2012 – May 2013 | April 2016 - March 2018 |
| **Trial completion** | January 2016 | June 2018 | October 2023 |
| **Key inclusion criteria** | - STEMI; - Primary PCI within 24 hours of symptom onset; - Presence of ≥1 acute infarct artery target vessel with ≥1 coronary artery stenosis in a native coronary artery that can be covered with ≥1 coronary stents; - Reference vessel diameter ≥2.25 mm and ≤4.0 mm. | - Symptomatic coronary artery disease including patients with chronic stable angina, silent ischemia, and acute coronary syndromes including non–ST-elevation and ST-elevation myocardial infarction; - Presence of ≥1 coronary artery stenosis >50% in a native coronary artery or a saphenous bypass graft in a vessel, which can be treated with a stent ranging in diameter from 2.25 to 4.0 mm and can be covered with 1 or multiple stents | - STEMI; - Primary PCI within 24 hours of symptom onset; - Presence of ≥1 acute infarct artery target vessel with ≥1 coronary artery stenosis in a native coronary artery that can be covered with ≥1 coronary stents; - Reference vessel diameter ≥2.25 mm and ≤4.0 mm. |
| **Key exclusion criteria** | - Known intolerance to aspirin, clopidogrel, heparin, stainless steel, biolimus, or contrast material; - Non-cardiac comorbid conditions with life expectancy of less than 1 year; - Mechanical complication of acute myocardial infarction; - Acute myocardial infarction due to stent thrombosis; - Planned surgery within 6 m of PCI unless dual-antiplatelet therapy is maintained throughout the perisurgical period; - Use of vitamin K antagonist. | - Known intolerance to aspirin, clopidogrel, heparin, stainless steel, sirolimus, everolimus, or contrast material; - Planned surgery within 6 m of PCI unless dual-antiplatelet therapy is maintained throughout the perisurgical period. | - Known intolerance to aspirin, ticagrelor, prasugrel, clopidogrel, sirolimus, everolimus, or contrast media; - Non-cardiac comorbid conditions with life expectancy of less than 1 year; - Mechanical complication of acute myocardial infarction; - Acute myocardial infarction due to stent thrombosis; - Planned surgery within 6 m of PCI unless dual-antiplatelet therapy is maintained throughout the perisurgical period. |
| **Definition of STEMI** | - New, or presumed new, persistent ST-segment elevation ≥1 mm in ≥2 contiguous leads; or - New, or presumed new, left bundle branch block; or - True posterior MI | - New, or presumed new, persistent ST-segment elevation ≥1 mm in ≥2 contiguous leads; or - New, or presumed new, left bundle branch block; or - New, or presumed new, horizontal or down-sloping ST-segment depression ≥1 mm in leads V1-V3. | - New, or presumed new, persistent ST-segment elevation ≥1 mm in ≥2 contiguous leads; or - New, or presumed new, left bundle branch block; or - New, or presumed new, horizontal or down-sloping ST-segment depression ≥1 mm in leads V1-V3. |
| **End point definition** |  |  |  |
| **Death** | Cardiac death: All deaths are considered cardiac unless an unequivocal non-cardiac cause can be established.  Vascular death: Death caused by non-coronary vascular causes, such as cerebrovascular disease, pulmonary embolism, ruptured aortic aneurysm, dissecting aneurysm, or other vascular cause.  Non-cardiovascular death: Any death not covered by the above definitions, including death due to infection, sepsis, pulmonary causes, accident, suicide or trauma. | All deaths are considered cardiac unless an unequivocal non-cardiac cause can be established.  Cardiac death: Any death due to immediate cardiac cause (e.g. MI, low-output failure, fatal arrhythmia). Unwitnessed death and death of unknown cause will be classified as cardiac death.  Vascular death: Death due to cerebrovascular disease, pulmonary embolism, ruptured aortic aneurysm, dissecting aneurysm, or other vascular cause.  Non-cardiovascular death: Any death not covered by the above definitions, including death due to infection, sepsis, pulmonary causes, accident, suicide or trauma. | All deaths are considered cardiac unless an unequivocal non-cardiac cause can be established.  Cardiac death: Any death due to immediate cardiac cause (e.g. MI, low-output failure, fatal arrhythmia). Unwitnessed death and death of unknown cause will be classified as cardiac death.  Vascular death: Death due to cerebrovascular disease, pulmonary embolism, ruptured aortic aneurysm, dissecting aneurysm, or other vascular cause.  Non-cardiovascular death: Any death not covered by the above definitions, including death due to infection, sepsis, pulmonary causes, accident, suicide or trauma. |
| **Stroke** | Rapidly developed clinical signs of focal or global disturbance of cerebral function lasting >24 hours with imaging of an acute clinically relevant brain lesion. Ischaemic cerebral infarctions with conversion to haemorrhage are listed here. The diagnosis of a intracerebral haemorrhage must be confirmed by cerebral imaging. | Ischaemic stroke, intracerebral haemorrhagic stroke, and cerebrovascular events with unclear cause. | Ischaemic stroke, intracerebral haemorrhagic stroke, and cerebrovascular events with unclear cause. |
| **Spontaneous MI** | Spontaneous Q-wave MI (>48 hours after index intervention):  The diagnosis of Q-wave myocardial infarction requires new pathological Q-waves in two or more contiguous ECG leads. Elevated cardiac biomarkers or a history of chest pain or other acute symptoms consistent with myocardial ischaemia are supportive of the diagnosis and should be documented but not needed to make the diagnosis (silent myocardial infarction).  Spontaneous non-Q-wave MI (>48 hours after index intervention):  Appropriate cardiac enzyme data (respecting top down hierarchy): 1) CK-MB >1*URL, or 2) in the absence of CK-MB, standard troponin >1*URL (high sensitivity troponin is considered as missing troponin), or 3) in the absence of troponin and CK-MB: CEC decision upon clinical scenario and with guidance of high sensitivity troponin. The appropriate enzyme data should be considered in conjunction with at least one of the following parameters: 1) evidence of myocardial ischaemia as recognised by clinical symptoms, or 2) evidence of myocardial ischaemia as recognised by electrocardiographic changes, or 3) evidence of myocardial ischaemia as recognised by cardiac imaging of new loss of viable myocardium or new regional wall motion abnormality. | Either one of the following criteria satisfies the diagnosis for an acute, evolving or recent MI:  1. Typical rise and gradual fall (Troponin) or more rapid rise and fall (CK-MB) of biochemical markers of myocardial necrosis with at least one of the following:  a. ischemic symptoms;  b. development of new pathologic (defined by Minnesota Code) Q-waves on the ECG;  c. ECG changes indicative of ischemia (ST segment elevation or depression);  2. Pathologic findings of an acute MI.  3. Development of new pathologic Q-waves on follow-up ECG in the absence of cardiac biomarker assessment during the acute event. | Either one of the following criteria satisfies the diagnosis for an acute, evolving or recent MI:  1. Typical rise and gradual fall (Troponin) or more rapid rise and fall (CK-MB) of biochemical markers of myocardial necrosis with at least one of the following:  a. ischemic symptoms;  b. development of new pathologic (defined by Minnesota Code) Q-waves on the ECG;  c. ECG changes indicative of ischemia (ST segment elevation or depression);  2. Pathologic findings of an acute MI.  3. Development of new pathologic Q-waves on follow-up ECG in the absence of cardiac biomarker assessment during the acute event. |
| CK-MB = creatine kinase-myocardial band; ECG = electrocardiogram; MI = myocardial infarction; PCI = percutaneous coronary intervention; STEMI = ST-segment elevation myocardial infarction; URL = upper reference limit. | | | |

# **Supplementary Table S1. Prescription of GDMT classes after STEMI among patients with available medication information**

|  | **GDMT class** | | | | |
| --- | --- | --- | --- | --- | --- |
|  | **4 class** | **3 class** | **2 class** | **1 class** | **0 class** |
| **COMFORTABLE-AMI** |  |  |  |  |  |
| Discharge | 398/595 [66.9%] | 174/595 [29.2%] | 22/595 [3.7%] | 1/595 [0.2%] | 0/595 [ 0%] |
| 1 year | 332/565 [58.8%] | 176/565 [31.2%] | 49/565 [8.7%] | 6/565 [1.1%] | 2/565 [0.4%] |
| 2 years | 319/552 [57.8%] | 171/552 [31.0%] | 50/552 [9.1%] | 10/552 [1.8%] | 2/552 [0.4%] |
| 5 years | 276/520 [53.1%] | 176/520 [33.9%] | 48/520 [9.2%] | 9/520 [1.7%] | 11/520 [2.1%] |
| **BIOSCIENCE** |  |  |  |  |  |
| Discharge | 339/406 [83.5%] | 60/406 [14.8%] | 7/406 [1.7%] | 0/406 [0%] | 0/406 [0%] |
| 1 year | 259/379 [68.3%] | 98/379 [25.9%] | 17/379 [4.5%] | 4/379 [1.1%] | 1/379 [0.3%] |
| 2 years | 206/372 [55.4%] | 120/372 [32.3%] | 39/372 [10.5%] | 7/372 [1.9%] | 0/372 [0%] |
| 5 years | 169/338 [50.0%] | 123/338 [36.4%] | 36/338 [10.7%] | 9/338 [2.7%] | 1/338 [0.3%] |
| **BIOSTEMI** |  |  |  |  |  |
| Discharge | 940/1269 [74.1%] | 274/1269 [21.6%] | 49/1269 [3.9%] | 6/1269 [0.5%] | 0/1269 [0%] |
| 1 year | 696/1188 [58.6%] | 348/1188 [29.3%] | 121/1188 [10.2%] | 21/1188 [1.8%] | 2/1188 [0.2%] |
| 2 years | 564/1161 [48.6%] | 394/1161 [33.9%] | 164/1161 [14.1%] | 32/1161 [2.8%] | 7/1161 [0.6%] |
| 5 years | 408/1008 [40.5%] | 361/1008 [35.8%] | 197/1008 [19.5%] | 36/1008 [3.6%] | 6/1008 [0.6%] |
| **Real-world PCI registry** |  |  |  |  |  |
| Discharge | 4606/5643 [81.6%] | 830/5643 [14.7%] | 175/5643 [3.1%] | 27/5643 [0.5%] | 5/5643 [0.1%] |
| 1 year | 3170/4906 [64.6%] | 1261/4906 [25.7%] | 359/4906 [7.3%] | 97/4906 [2.0%] | 19/4906 [0.4%] |
| Depicted are counts / sample size [%].  GDMT = guideline-directed medical therapy; PCI = percutaneous coronary intervention; STEMI = ST-segment elevation myocardial infarction. | | | | | |

# **Supplementary Table S2. Longitudinal distribution of GDMT classes after STEMI including death and unavailable medication information**

|  | **GDMT class** | | | | | **Patient deceased** | **No information** |
| --- | --- | --- | --- | --- | --- | --- | --- |
|  | **4 class** | **3 class** | **2 class** | **1 class** | **0 class** |  |  |
| **COMFORTABLE-AMI** |  |  |  |  |  |  |  |
| Discharge | 398/595 [66.9%] | 174/595 [29.2%] | 22/595 [3.7%] | 1/595 [0.2%] | 0/595 [0%] | - | - |
| 1 year | 332/595 [55.8%] | 176/595 [29.6%] | 49/595 [8.2%] | 6/595 [1%] | 2/595 [0.3%] | 14/595 [2.4%] | 16/595 [2.7%] |
| 2 years | 319/595 [53.6%] | 171/595 [28.7%] | 50/595 [8.4%] | 10/595 [1.7%] | 2/595 [0.3%] | 22/595 [3.7%] | 21/595 [3.5%] |
| 5 years | 276/595 [46.4%] | 176/595 [29.6%] | 48/595 [8.1%] | 9/595 [1.5%] | 11/595 [1.8%] | 48/595 [8.1%] | 27/595 [4.5%] |
| **BIOSCIENCE** |  |  |  |  |  |  |  |
| Discharge | 339/406 [83.5%] | 60/406 [14.8%] | 7/406 [1.7%] | 0/406 [0%] | 0/406 [0%] | - | - |
| 1 year | 259/406 [63.8%] | 98/406 [24.1%] | 17/406 [4.2%] | 4/406 [1%] | 1/406 [0.2%] | 14/406 [3.4%] | 13/406 [3.2%] |
| 2 years | 206/406 [50.7%] | 120/406 [29.6%] | 39/406 [9.6%] | 7/406 [1.7%] | 0/406 [0%] | 18/406 [4.4%] | 16/406 [3.9%] |
| 5 years | 169/406 [41.6%] | 123/406 [30.3%] | 36/406 [8.9%] | 9/406 [2.2%] | 1/406 [0.2%] | 39/406 [9.6%] | 29/406 [7.1%] |
| **BIOSTEMI** |  |  |  |  |  |  |  |
| Discharge | 940/1269 [74.1%] | 274/1269 [21.6%] | 49/1269 [3.9%] | 6/1269 [0.5%] | 0/1269 [0%] | - | - |
| 1 year | 696/1269 [54.8%] | 348/1269 [27.4%] | 121/1269 [9.5%] | 21/1269 [1.7%] | 2/1269 [0.2%] | 24/1269 [1.9%] | 57/1269 [4.5%] |
| 2 years | 564/1269 [44.4%] | 394/1269 [31%] | 164/1269 [12.9%] | 32/1269 [2.5%] | 7/1269 [0.6%] | 30/1269 [2.4%] | 78/1269 [6.1%] |
| 5 years | 408/1269 [32.2%] | 361/1269 [28.4%] | 197/1269 [15.5%] | 36/1269 [2.8%] | 6/1269 [0.5%] | 69/1269 [5.4%] | 192/1269 [15.1%] |
| **Real-world PCI registry** |  |  |  |  |  |  |  |
| Discharge | 4606/5643 [81.6%] | 830/5643 [14.7%] | 175/5643 [3.1%] | 27/5643 [0.5%] | 5/5643 [0.1%] | - | - |
| 1 year | 3170/5643 [56.2%] | 1261/5643 [22.3%] | 359/5643 [6.4%] | 97/5643 [1.7%] | 19/5643 [0.3%] | 266/5643 [4.7%] | 471/5643 [8.3%] |
| Depicted are counts / sample size [%]. No information includes withdrawal of consent, lost-to-follow-up or medication information could not be retrieved.  GDMT = guideline-directed medical therapy; PCI = percutaneous coronary intervention; STEMI = ST-segment elevation myocardial infarction. | | | | | | | |

# **Supplementary Table S3.** **Temporal trends in prescription GDMT classes in the real-world PCI registry**

|  | **GDMT class at discharge** | | | | | **GDMT class at 1 year** | | | | |
| --- | --- | --- | --- | --- | --- | --- | --- | --- | --- | --- |
| **Year of index PCI** | **4 class** | **3 class** | **2 class** | **1 class** | **0 class** | **4 class** | **3 class** | **2 class** | **1 class** | **0 class** |
| **2009** | 255 / 290  [87.93%] | 29 / 290  [10%] | 6 / 290  [2.07%] | 0 / 290  [0%] | 0 / 290  [0%] | 161 / 258  [62.40%] | 72 / 258  [27.91%] | 18 / 258  [6.98%] | 7 / 258  [2.71%] | 0 / 258  [0%] |
| **2010** | 245 / 293  [83.62%] | 39 / 293  [13.31%] | 6 / 293  [2.05%] | 2 / 293  [0.68%] | 1 / 293  [0.34%] | 170 / 249  [68.27%] | 58 / 249  [23.29%] | 15 / 249  [6.02%] | 5 / 249  [2.01%] | 1 / 249  [0.40%] |
| **2011** | 345 / 427  [80.80%] | 65 / 427  [15.22%] | 13 / 427  [3.04%] | 1 / 427  [0.23%] | 3 / 427  [0.70%] | 243 / 364  [66.76%] | 94 / 364  [25.82%] | 15 / 364  [4.12%] | 7 / 364  [1.92%] | 5 / 364  [1.37%] |
| **2012** | 374 / 450  [83.11%] | 62 / 450  [13.78%] | 11 / 450  [2.44%] | 3 / 450  [0.67%] | 0 / 450  [0%] | 258 / 393  [65.65%] | 101 / 393  [25.70%] | 16 / 393  [4.07%] | 13 / 393  [3.31%] | 5 / 393  [1.27%] |
| **2013** | 301 / 391  [76.98%] | 70 / 391  [17.90%] | 15 / 391  [3.84%] | 4 / 391  [1.02%] | 1 / 391  [0.26%] | 217 / 332  [65.36%] | 80 / 332  [24.10%] | 22 / 332  [6.63%] | 13 / 332  [3.92%] | 0 / 332  [0%] |
| **2014** | 302 / 398  [75.88%] | 73 / 398  [18.34%] | 20 / 398  [5.03%] | 3 / 398  [0.75%] | 0 / 398  [0%] | 221 / 346  [63.87%] | 87 / 346  [25.14%] | 30 / 346  [8.67%] | 8 / 346  [2.31%] | 0 / 346  [0%] |
| **2015** | 312 / 397  [78.59%] | 69 / 397  [17.38%] | 13 / 397  [3.27%] | 3 / 397  [0.76%] | 0 / 397  [0%] | 222 / 350  [63.43%] | 95 / 350  [27.14%] | 20 / 350  [5.71%] | 12 / 350  [3.43%] | 1 / 350  [0.29%] |
| **2016** | 372 / 467  [79.66%] | 75 / 467  [16.06%] | 16 / 467  [3.43%] | 4 / 467  [0.86%] | 0 / 467  [0%] | 255 / 399  [63.91%] | 106 / 399  [26.57%] | 33 / 399  [8.27%] | 5 / 399  [1.25%] | 0 / 399  [0%] |
| **2017** | 382 / 474  [80.59%] | 68 / 474  [14.35%] | 21 / 474  [4.43%] | 3 / 474  [0.63%] | 0 / 474  [0%] | 278 / 408  [68.14%] | 97 / 408  [23.77%] | 29 / 408  [7.11%] | 4 / 408  [0.98%] | 0 / 408  [0%] |
| **2018** | 368 / 438  [84.02%] | 60 / 438  [13.70%] | 8 / 438  [1.83%] | 2 / 438  [0.46%] | 0 / 438  [0%] | 254 / 373  [68.10%] | 79 / 373  [21.18%] | 35 / 373  [9.38%] | 3 / 373  [0.80%] | 2 / 373  [0.54%] |
| **2019** | 403 / 454  [88.77%] | 40 / 454  [8.81%] | 11 / 454  [2.42%] | 0 / 454  [0%] | 0 / 454  [0%] | 248 / 397  [62.47%] | 105 / 397  [26.45%] | 32 / 397  [8.06%] | 12 / 397  [3.02%] | 0 / 397  [0%] |
| **2020** | 372 / 445  [83.60%] | 60 / 445  [13.48%] | 13 / 445  [2.92%] | 0 / 445  [0%] | 0 / 445  [0%] | 247 / 398  [62.06%] | 111 / 398  [27.89%] | 33 / 398  [8.29%] | 4 / 398  [1.01%] | 3 / 398  [0.75%] |
| **2021** | 371 / 457  [81.18%] | 70 / 457  [15.32%] | 15 / 457  [3.28%] | 1 / 457  [0.22%] | 0 / 457  [0%] | 269 / 422  [63.74%] | 112 / 422  [26.54%] | 38 / 422  [9.00%] | 2 / 422  [0.47%] | 1 / 422  [0.24%] |
| **2022** | 204 / 262  [77.86%] | 50 / 262  [19.08%] | 7 / 262  [2.67%] | 1 / 262  [0.38%] | 0 / 262  [0%] | 127 / 217  [58.53%] | 64 / 217  [29.49%] | 23 / 217  [10.60%] | 2 / 217  [0.92%] | 1 / 217  [0.46%] |
| Depicted are counts / sample size [%].  GDMT = guideline-directed medical therapy; PCI = percutaneous coronary intervention. | | | | | | | | | | |

# **Supplementary Table S4. Prescription of GDMT components after STEMI over time**

|  | **GDMT component** | | | | | | | | |
| --- | --- | --- | --- | --- | --- | --- | --- | --- | --- |
|  | **Any antithrombotic therapy** | **Aspirin** | **P2Y12 inhibitor** | **Oral anticoagulant** | **Statin or other lipid lowering therapy** | **Statin** | **Other lipid lowering therapy** | **Renin-angiotensin system inhibitor** | **Beta-blocker** |
| **COMFORTABLE-AMI** |  |  |  |  |  |  |  |  |  |
| Discharge | 595/595  [100%] | 594/595  [99.8%] | 594/595  [99.8%] | 22/595  [3.7%] | 588/594  [99.0%] | 584/594  [98.3%] | 22/594  [3.7%] | 464/594  [78.1%] | 512/594  [86.2%] |
| 1 year | 563/565  [99.7%] | 552/565  [97.7%] | 469/565  [83.0%] | 19/564  [3.4%] | 522/564  [92.6%] | 519/564  [92.0%] | 12/564  [2.1%] | 421/564  [74.7%] | 454/564  [80.5%] |
| 2 years | 546/552  [98.9%] | 532/552  [96.4%] | 89/552  [16.1%] | 26/552  [4.7%] | 512/552  [92.8%] | 507/552  [91.9%] | 21/552  [3.8%] | 408/552  [73.9%] | 433/552  [78.4%] |
| 5 years | 505/520  [97.1%] | 471/520  [90.6%] | 53/520  [10.2%] | 36/520  [6.9%] | 463/520  [89.0%] | 448/520  [86.2%] | 34/520  [6.5%] | 388/520  [74.6%] | 381/520  [73.3%] |
| **BIOSCIENCE** |  |  |  |  |  |  |  |  |  |
| Discharge | 406/406  [100%] | 404/406  [99.5%] | 404/406  [99.5%] | 15/406  [3.7%] | 397/406  [97.8%] | 397/406  [97.8%] | 3/406  [0.7%] | 383/406  [94.3%] | 364/406  [89.7%] |
| 1 year | 378/379  [99.7%] | 369/379  [97.4%] | 283/379  [74.7%] | 17/379  [4.5%] | 359/379  [94.7%] | 357/378  [94.4%] | 14/379  [3.7%] | 317/379  [83.6%] | 314/379  [82.9%] |
| 2 years | 370/372  [99.5%] | 358/372  [96.2%] | 48/372  [12.9%] | 19/372  [5.11] | 343/372  [92.2%] | 343/372  [92.2%] | 19/372  [5.1%] | 290/372  [78.0%] | 266/372  [71.5%] |
| 5 years | 337/338  [99.7%] | 304/338  [89.9%] | 31/338  [9.2%] | 29/338  [8.6%] | 303/338  [89.6%] | 297/338  [87.9%] | 47/338  [13.9%] | 256/338  [75.7%] | 230/338  [68.1%] |
| **BIOSTEMI** |  |  |  |  |  |  |  |  |  |
| Discharge | 1269/1269  [100%] | 1266/1269  [99.8%] | 1265/1269  [99.7%] | 73/1269  [5.8%] | 1240/1269  [97.7%] | 1231/1269  [97.0%] | 56/1269  [4.4%] | 1159/1269  [91.3%] | 1018/1269  [80.2%] |
| 1 year | 1183/1188  [99.6%] | 1141/1188  [96.0%] | 730/1188  [61.5%] | 84/1188  [7.1%] | 1093/1188  [92.0%] | 1076/1188  [90.6%] | 147/1188  [12.4%] | 957/1188  [80.6%] | 858/1188  [72.2%] |
| 2 years | 1142/1161  [98.4%] | 1067/1161  [91.9%] | 106/1161  [9.1%] | 96/1161  [8.3%] | 1046/1161  [90.1%] | 1015/1161  [87.4%] | 198/1161  [17.1%] | 875/116  [75.4%] | 735/1161  [63.3%] |
| 5 years | 990/1008  [98.2%] | 891/1008  [88.4%] | 79/1008  [7.4%] | 111/1008  [11.0%] | 880/1008  [87.3%] | 840/1008  [83.3%] | 305/1008  [30.3%] | 700/1008  [69.4%] | 575/1008  [57.0%] |
| **Real-world PCI registry** |  |  |  |  |  |  |  |  |  |
| Discharge | 5637/5643  [99.9%] | 5597/5643  [99.2%] | 5607/5643  [99.4%] | 416/5642  [7.4%] | 5360/5642  [95.0%] | 5348/5642  [94.8%] | 151/5642  [2.7%] | 5265/5642  [93.3%] | 5029/5642  [89.1%] |
| 1 year | 4876/4907  [99.4%] | 4518/4907  [92.1%] | 3966/4907  [80.8%] | 543/4906  [11.1%] | 4443/4906  [90.6%] | 4376/4905  [89.2%] | 732/4906  [14.9%] | 4026/4906  [82.1%] | 3935/4905  [80.2%] |
| Depicted are counts / sample size (%).  GDMT = guideline-directed medical therapy; PCI = percutaneous coronary intervention; STEMI = ST-segment elevation myocardial infarction. | | | | | | | | | |

# **Supplementary Table S5. Univariable Cox proportional hazards models for clinical outcomes in the pooled randomised cohort**

|  | **GDMT class 4 vs. 0-2** | | **GDMT class 3 vs. 0-2** | | **GDMT class 4 vs. 3** | |
| --- | --- | --- | --- | --- | --- | --- |
|  | **HR (95%CI)** | **P value** | **HR (95%CI)** | **P value** | **HR (95%CI)** | **P value** |
| **At 30 days** |  |  |  |  |  |  |
| Composite of CV death, stroke, or myocardial infarction, n (%) | 0.28 (0.15-0.53) | <0.001 | 0.16 (0.06-0.43) | <0.001 | 1.77 (0.93-3.36) | 0.081 |
| Cardiovascular death, n (%) | 0.21 (0.06-0.72) | 0.013 | 0.11 (0.03-0.43) | 0.002 | 1.98 (0.42-9.36) | 0.391 |
| Myocardial infarction, (%) | 0.75 (0.17-3.36) | 0.702 | 0.33 (0.24-0.45) | <0.001 | 2.28 (0.37-13.92) | 0.371 |
| Stroke, n (%) | 0.15 (0.00-6.26) | 0.317 | 0.16 (0.00-6.18) | 0.327 | 0.91 (0.66-1.27) | 0.587 |
| All-cause death, n (%) | 0.23 (0.08-0.66) | 0.006 | 0.16 (0.05-0.56) | 0.004 | 1.42 (0.76-2.64) | 0.269 |
| **At 1 year** |  |  |  |  |  |  |
| Composite of CV death, stroke, or myocardial infarction, n (%) | 0.40 (0.34-0.47) | <0.001 | 0.40 (0.18-0.91) | 0.030 | 0.99 (0.45-2.17) | 0.978 |
| Cardiovascular death, n (%) | 0.29 (0.07-1.15) | 0.077 | 0.43 (0.07-2.58) | 0.359 | 0.66 (0.25-1.78) | 0.414 |
| Myocardial infarction, (%) | 0.91 (0.26-3.15) | 0.883 | 0.39 (0.29-0.54) | <0.001 | 2.32 (0.49-10.97) | 0.288 |
| Stroke, n (%) | 0.21 (0.08-0.54) | 0.001 | 0.46 (0.09-2.49) | 0.369 | 0.46 (0.22-0.96) | 0.038 |
| All-cause death, n (%) | 0.32 (0.11-0.96) | 0.042 | 0.41 (0.13-1.32) | 0.135 | 0.79 (0.37-1.69) | 0.549 |
| **At 5 years** |  |  |  |  |  |  |
| Composite of CV death, stroke, or myocardial infarction, n (%) | 0.42 (0.27-0.64) | <0.001 | 0.38 (0.20-0.74) | 0.004 | 1.10 (0.86-1.41) | 0.437 |
| Cardiovascular death, n (%) | 0.33 (0.16-0.68) | 0.002 | 0.29 (0.07-1.14) | 0.076 | 1.15 (0.59-2.24) | 0.679 |
| Myocardial infarction, (%) | 0.64 (0.56-0.73) | 0.000 | 0.52 (0.37-0.73) | <0.001 | 1.24 (0.89-1.74) | 0.201 |
| Stroke, n (%) | 0.39 (0.14-1.10) | 0.074 | 0.36 (0.07-1.97) | 0.238 | 1.08 (0.50-2.35) | 0.843 |
| All-cause death, n (%) | 0.42 (0.22-0.82) | 0.011 | 0.38 (0.16-0.93) | 0.033 | 1.09 (0.87-1.38) | 0.447 |
| Cox proportional hazards models were used to estimate hazard ratios (HRs) with 95% confidence intervals (CIs). Models were adjusted for clustering by trial.  CV = cardiovascular; GDMT = guideline-directed medical therapy; MI = myocardial infarction. | | | | | | |

# **Supplementary Table S6. Exploratory analysis of the composite outcome according to specific GDMT class 3 combinations**

|  | **Events/patients, n/N (%)** | **Hazard ratio (95%CI)** | **P value** | **Adjusted Hazard ratio (95%CI)** | **Adjusted P value** |
| --- | --- | --- | --- | --- | --- |
| **Pooled randomised cohort** |  |  |  |  |  |
| **At 30 days** |  |  |  |  |  |
| GDMT 4 class | 29/1677 (1.7%) | Ref. |  | Ref. |  |
| GDMT 3 class: any antithrombotic therapy, lipid lowering therapy, beta-blocker | 3/183 (1.7%) | 1.06 (0.30-3.74) | 0.924 | Not estimated | Not estimated |
| GDMT 3 class: any antithrombotic therapy, lipid lowering therapy, and renin-angiotensin system inhibitor | 2/293 (0.7%) | 2.56 (0.74-8.82) | 0.137 | Not estimated | Not estimated |
| GDMT 3 class: any antithrombotic therapy, beta-blocker, and renin-angiotensin system inhibitor | 0/ 32 (0%) | Not estimated | Not estimated | Not estimated | Not estimated |
| GDMT 3 class: lipid lowering therapy, beta-blocker, and renin-angiotensin system inhibitor | NA/ 0 | Not estimated | Not estimated | Not estimated | Not estimated |
| **At 1 year** |  |  |  |  |  |
| GDMT 4 class | 68/1677 (4.1%) | Ref. |  | Ref. |  |
| GDMT 3 class: any antithrombotic therapy, lipid lowering therapy, beta-blocker | 9/183 (5.0%) | 0.83 (0.27-2.54) | 0.746 | 0.53 (0.14-2.02) | 0.348 |
| GDMT 3 class: any antithrombotic therapy, lipid lowering therapy, and renin-angiotensin system inhibitor | 9/293 (3.1%) | 1.34 (0.92-1.96) | 0.131 | 1.12 (0.79-1.59) | 0.527 |
| GDMT 3 class: any antithrombotic therapy, beta-blocker, and renin-angiotensin system inhibitor | 3/ 32 (10.0%) | 0.41 (0.07-2.52) | 0.336 | 0.38 (0.10-1.39) | 0.145 |
| GDMT 3 class: lipid lowering therapy, beta-blocker, and renin-angiotensin system inhibitor | NA/0 | Not estimated | Not estimated | Not estimated | Not estimated |
| **At 5 years** |  |  |  |  |  |
| GDMT 4 class | 181/1677 (11.7%) | Ref. |  | Ref. |  |
| GDMT 3 class: any antithrombotic therapy, lipid lowering therapy, beta-blocker | 25/183 (14.3%) | 0.80 (0.52-1.23) | 0.307 | 0.63 (0.40-0.99) | 0.046 |
| GDMT 3 class: any antithrombotic therapy, lipid lowering therapy, and renin-angiotensin system inhibitor | 20/293 (7.1%) | 1.66 (1.15-2.38) | 0.006 | 1.39 (1.12-1.74) | 0.004 |
| GDMT 3 class: any antithrombotic therapy, beta-blocker, and renin-angiotensin system inhibitor | 6/ 32 (21.7%) | 0.52 (0.25-1.09) | 0.084 | 0.49 (0.41-0.59) | <0.001 |
| GDMT 3 class: lipid lowering therapy, beta-blocker, and renin-angiotensin system inhibitor | NA/0 | Not estimated | Not estimated | Not estimated | Not estimated |
| **Bern PCI registry** |  |  |  |  |  |
| **At 30 days** |  |  |  |  |  |
| GDMT 4 class | 139/4606 (3.1%) | Ref. |  | Ref. |  |
| GDMT 3 class: any antithrombotic therapy, lipid lowering therapy, beta-blocker | 14/207 (6.9%) | 0.45 (0.26-0.77) | 0.004 | 0.51 (0.26-0.98) | 0.043 |
| GDMT 3 class: any antithrombotic therapy, lipid lowering therapy, and renin-angiotensin system inhibitor | 21/438 (5.1%) | 0.62 (0.39-0.98) | 0.041 | 0.77 (0.43-1.37) | 0.371 |
| GDMT 3 class: any antithrombotic therapy, beta-blocker, and renin-angiotensin system inhibitor | 15/184 (8.6%) | 0.36 (0.21-0.61) | <0.001 | 0.52 (0.26-1.02) | 0.057 |
| GDMT 3 class: lipid lowering therapy, beta-blocker, and renin-angiotensin system inhibitor | 0/1 (0%) | Not estimated | Not estimated | Not estimated | Not estimated |
| **At 1 year** |  |  |  |  |  |
| GDMT 4 class | 264/4606 (6.1%) | Ref. |  | Ref. |  |
| GDMT 3 class: any antithrombotic therapy, lipid lowering therapy, beta-blocker | 18/207 (8.9%) | 0.66 (0.41-1.06) | 0.086 | 0.72 (0.42-1.24) | 0.239 |
| GDMT 3 class: any antithrombotic therapy, lipid lowering therapy, and renin-angiotensin system inhibitor | 39/438 (9.5%) | 0.63 (0.45-0.88) | 0.007 | 0.81 (0.53-1.24) | 0.334 |
| GDMT 3 class: any antithrombotic therapy, beta-blocker, and renin-angiotensin system inhibitor | 25/184 (14.6%) | 0.40 (0.26-0.60) | <0.001 | 0.57 (0.34-0.94) | 0.029 |
| GDMT 3 class: lipid lowering therapy, beta-blocker, and renin-angiotensin system inhibitor | 0/1 (0%) | Not estimated | Not estimated | Not estimated | Not estimated |
| Kaplan–Meier estimates were used to calculate cumulative event rates, and Cox proportional hazards models were used to estimate hazard ratios (HRs) with 95% confidence intervals (CIs). For the pooled randomised cohort, standard errors were adjusted for clustering by trial. Only the first occurrence of each event type was considered for each patient.  GDMT = guideline-directed medical therapy. | | | | | |

# **Supplementary Table S7. Clinical outcomes stratified by GDMT class at discharge in the real-world PCI registry**

|  | **GDMT 4 class at discharge**  **N = 4,606** | **GDMT 3 class at discharge**  **N = 830** | **GDMT 0-2 class at discharge**  **N = 207** | **GDMT class 4 vs. 0-2** | | **GDMT class 3 vs. 0-2** | | **GDMT class 4 vs. 3** | |
| --- | --- | --- | --- | --- | --- | --- | --- | --- | --- |
|  |  |  |  | **Adjusted HR (95%CI)** | **Adjusted P value** | **Adjusted HR (95%CI)** | **Adjusted P value** | **Adjusted HR (95%CI)** | **Adjusted P value** |
| **At 30 days** |  |  |  |  |  |  |  |  |  |
| Composite of CV death, stroke, or myocardial infarction, n (%) | 139 (3.1%) | 50 (6.3%) | 38 (19.1%) | 0.29 (0.17-0.47) | <0.001 | 0.45 (0.26-0.79) | 0.005 | 0.64 (0.42-0.96) | 0.029 |
| Cardiovascular death, n (%) | 56 (1.3%) | 26 (3.3%) | 29 (14.7%) | 0.17 (0.09-0.32) | <0.001 | 0.33 (0.16-0.66) | 0.002 | 0.52 (0.29-0.94) | 0.030 |
| Myocardial infarction, (%) | 62 (1.4%) | 8 (1.0%) | 3 (1.5%) | 0.85 (0.20-3.58) | 0.820 | 0.55 (0.11-2.85) | 0.475 | 1.54 (0.60-3.96) | 0.368 |
| Stroke, n (%) | 29 (0.7%) | 19 (2.4%) | 6 (3.1%) | 0.51 (0.15-1.76) | 0.289 | 1.24 (0.35-4.38) | 0.744 | 0.41 (0.21-0.83) | 0.014 |
| All-cause death, n (%) | 66 (1.5%) | 28 (3.6%) | 33 (16.6%) | 0.17 (0.10-0.31) | <0.001 | 0.30 (0.16-0.58) | <0.001 | 0.57 (0.33-1.00) | 0.048 |
| **At 1 year** |  |  |  |  |  |  |  |  |  |
| Composite of CV death, stroke, or myocardial infarction, n (%) | 264 (6.1%) | 82 (10.5%) | 48 (24.5%) | 0.41 (0.27-0.62) | <0.001 | 0.55 (0.34-0.89) | 0.015 | 0.73 (0.54-1.00) | 0.049 |
| Cardiovascular death, n (%) | 110 (2.6%) | 51 (6.6%) | 37 (19.1%) | 0.28 (0.17-0.47) | <0.001 | 0.54 (0.31-0.96) | 0.034 | 0.52 (0.34-0.78) | 0.002 |
| Myocardial infarction, (%) | 117 (2.7%) | 15 (2.0%) | 5 (2.8%) | 1.53 (0.37-6.27) | 0.557 | 0.82 (0.17-3.86) | 0.799 | 1.87 (0.90-3.89) | 0.095 |
| Stroke, n (%) | 55 (1.3%) | 23 (2.9%) | 8 (4.3%) | 0.47 (0.18-1.21) | 0.119 | 0.79 (0.29-2.18) | 0.651 | 0.59 (0.33-1.07) | 0.081 |
| All-cause death, n (%) | 156 (3.6%) | 62 (8.0%) | 48 (24.5%) | 0.27 (0.17-0.41) | <0.001 | 0.41 (0.25-0.66) | <0.001 | 0.65 (0.45-0.93) | 0.017 |
| Kaplan–Meier estimates were used to calculate cumulative event rates, and Cox proportional hazards models were used to estimate hazard ratios (HRs) with 95% confidence intervals (CIs). Only the first occurrence of each event type was considered for each patient.  CV = cardiovascular; GDMT = guideline-directed medical therapy; MI = myocardial infarction. | | | | | | | | | |

# **Supplementary Table S8. Univariable Cox proportional hazards models for clinical outcomes in the real-world PCI registry**

|  | **GDMT class 4 vs. 0-2** | | **GDMT class 3 vs. 0-2** | | **GDMT class 4 vs. 3** | |
| --- | --- | --- | --- | --- | --- | --- |
|  | **HR (95%CI)** | **P value** | **HR (95%CI)** | **P value** | **HR (95%CI)** | **P value** |
| **At 30 days** |  |  |  |  |  |  |
| Composite of CV death, stroke, or myocardial infarction, n (%) | 0.15 (0.11-0.22) | <0.001 | 0.15 (0.11-0.22) | <0.001 | 0.49 (0.36-0.68) | <0.001 |
| Cardiovascular death, n (%) | 0.08 (0.05-0.12) | <0.001 | 0.08 (0.05-0.12) | <0.001 | 0.38 (0.24-0.61) | <0.001 |
| Myocardial infarction, (%) | 0.89 (0.28-2.82) | 0.839 | 0.89 (0.28-2.82) | 0.506 | 1.39 (0.67-2.91) | 0.379 |
| Stroke, n (%) | 0.21 (0.09-0.50) | <0.001 | 0.21 (0.09-0.50) | 0.559 | 0.27 (0.15-0.48) | <0.001 |
| All-cause death, n (%) | 0.08 (0.05-0.12) | <0.001 | 0.08 (0.05-0.12) | <0.001 | 0.42 (0.27-0.65) | <0.001 |
| **At 1 year** |  |  |  |  |  |  |
| Composite of CV death, stroke, or myocardial infarction, n (%) | 0.21 (0.16-0.29) | <0.001 | 0.21 (0.16-0.29) | <0.001 | 0.57 (0.44-0.73) | <0.001 |
| Cardiovascular death, n (%) | 0.12 (0.08-0.17) | <0.001 | 0.12 (0.08-0.17) | <0.001 | 0.38 (0.27-0.53) | <0.001 |
| Myocardial infarction, (%) | 0.93 (0.38-2.27) | 0.866 | 0.93 (0.38-2.27) | 0.432 | 1.39 (0.81-2.38) | 0.230 |
| Stroke, n (%) | 0.27 (0.13-0.57) | 0.001 | 0.27 (0.13-0.57) | 0.292 | 0.42 (0.26-0.68) | <0.001 |
| All-cause death, n (%) | 0.12 (0.09-0.17) | <0.001 | 0.12 (0.09-0.17) | <0.001 | 0.44 (0.33-0.60) | <0.001 |
| Cox proportional hazards models were used to estimate hazard ratios (HRs) with 95% confidence intervals (CIs).  CV = cardiovascular; GDMT = guideline-directed medical therapy; MI = myocardial infarction. | | | | | | |

# **Supplementary Table S9. Proportional hazards assessment and piecewise Cox analyses for clinical outcomes in the real-world PCI registry**

|  | **P value for proportional hazards assumption** | **HR (95% CI)**  **GDMT class 4 vs. 0-2** | | **HR (95% CI)**  **GDMT class 3 vs. 0-2** | |
| --- | --- | --- | --- | --- | --- |
|  |  | **0-180 days** | **181-365 days** | **0-180 days** | **181-365 days** |
| Composite of CV death, stroke, or myocardial infarction | <0.001 | 0.18 (0.13-0.25) | 1.68 (0.23-12.1) | 0.33 (0.23-0.48) | 2.32 (0.30-17.9) |
| Cardiovascular death | <0.001 | 0.10 (0.07-0.15) | 0.66 (0.09-4.93) | 0.24 (0.15-0.37) | 2.59 (0.34-19.8) |
| Myocardial infarction | 0.34 | 0.81 (0.33-1.98) | Not estimated | 0.68 (0.25-1.86) | Not estimated |
| Stroke | 0.02 | 0.20 (0.10-0.44) | Not estimated | 0.64 (0.28-1.43) | Not estimated |
| All-cause death | <0.001 | 0.11 (0.08-0.15) | 0.38 (0.12-1.25) | 0.22 (0.15-0.33) | 1.13 (0.33-3.86) |
| Hazard ratios (HRs) and 95% confidence intervals (95% Cis) were not estimated when the number of events was insufficient for model estimation.  GDMT = guideline-directed medical therapy. | | | | | |

# **Supplementary Table S10.** **Sensitivity analysis: clinical outcomes stratified by GDMT class at discharge in the pooled randomised cohort (only post-discharge outcomes counted)**

|  | **GDMT 4 class at discharge**  **N =1,677** | **GDMT 3 class at discharge**  **N = 508** | **GDMT 0-2 class at discharge**  **N = 85** | **GDMT class 4 vs. 0-2** | | **GDMT class 3 vs. 0-2** | | **GDMT class 4 vs. 3** | |
| --- | --- | --- | --- | --- | --- | --- | --- | --- | --- |
|  |  |  |  | **Adjusted HR (95%CI)** | **Adjusted P value** | **Adjusted HR (95%CI)** | **Adjusted P value** | **Adjusted HR (95%CI)** | **Adjusted P value** |
| **At 30 days** |  |  |  |  |  |  |  |  |  |
| Composite of CV death, stroke, or myocardial infarction, n (%) | 23 (1.4%) | 4 (0.8%) | 4 (4.9%) | 0.46 (0.30-0.71) | <0.001 | 0.37 (0.21-0.66) | 0.001 | 1.23 (0.45-3.37) | 0.683 |
| Cardiovascular death, n (%) | 14 (0.8%) | 3 (0.6%) | 3 (3.6%) | 0.63 (0.33-1.20) | 0.164 | 0.59 (0.18-1.93) | 0.387 | 1.07 (0.17-6.57) | 0.946 |
| Myocardial infarction, (%) | 8 (0.5%) | 1 (0.2%) | 0 (0%) |  |  |  |  | 1.10 (0.59-2.06) | 0.761 |
| Stroke, n (%) | 2 (0.1%) | 1 (0.2%) | 1 (1.3%) | 0.14 (0.05-0.36) | <0.001 | 0.19 (0.10-0.38) | <0.001 | 0.72 (0.55-0.94) | 0.017 |
| All-cause death, n (%) | 14 (0.8%) | 3 (0.6%) | 3 (3.6%) | 0.63 (0.33-1.20) | 0.164 | 0.59 (0.18-1.93) | 0.387 | 1.07 (0.17-6.57) | 0.946 |
| **At 1 year** |  |  |  |  |  |  |  |  |  |
| Composite of CV death, stroke, or myocardial infarction, n (%) | 63 (3.8%) | 21 (4.2%) | 6 (7.4%) | 0.76 (0.38-1.52) | 0.437 | 1.06 (0.95-1.18) | 0.314 | 0.72 (0.32-1.60) | 0.419 |
| Cardiovascular death, n (%) | 25 (1.5%) | 12 (2.4%) | 4 (4.9%) | 1.40 (0.96-2.03) | 0.080 | 2.99 (1.38-6.44) | 0.005 | 0.47 (0.15-1.47) | 0.193 |
| Myocardial infarction, (%) | 31 (1.9%) | 5 (1.2%) | 1 (1.3%) | 1.04 (0.66-1.62) | 0.879 | 0.98 (0.91-1.06) | 0.641 | 1.05 (0.63-1.77) | 0.844 |
| Stroke, n (%) | 9 (0.6%) | 6 (1.2%) | 1 (1.3%) | 0.24 (0.19-0.32) | <0.001 | 0.39 (0.19-0.80) | 0.010 | 0.62 (0.23-1.65) | 0.341 |
| All-cause death, n (%) | 34 (2.1%) | 13 (2.6%) | 5 (6.1%) | 0.99 (0.54-1.81) | 0.972 | 1.52 (1.06-2.17) | 0.021 | 0.65 (0.25-1.70) | 0.380 |
| **At 5 years** |  |  |  |  |  |  |  |  |  |
| Composite of CV death, stroke, or myocardial infarction, n (%) | 179 (11.5%) | 52 (10.7%) | 17 (23.1%) | 0.62 (0.60-0.65) | <0.001 | 0.66 (0.60-0.72) | <0.001 | 0.94 (0.90-0.99) | 0.029 |
| Cardiovascular death, n (%) | 71 (4.6%) | 22 (4.5%) | 9 (12.1%) | 0.58 (0.25-1.33) | 0.200 | 0.82 (0.32-2.08) | 0.678 | 0.71 (0.64-0.78) | <0.001 |
| Myocardial infarction, (%) | 95 (6.2%) | 24 (5.2%) | 6 (9.3%) | 0.99 (0.43-2.29) | 0.981 | 0.92 (0.44-1.91) | 0.816 | 1.08 (0.98-1.20) | 0.140 |
| Stroke, n (%) | 35 (2.3%) | 10 (2.1%) | 3 (4.4%) | 0.33 (0.10-1.05) | 0.060 | 0.22 (0.03-1.56) | 0.130 | 1.49 (0.67-3.29) | 0.328 |
| All-cause death, n (%) | 113 (7.3%) | 33 (6.9%) | 12 (15.8%) | 0.80 (0.46-1.41) | 0.444 | 0.91 (0.36-2.30) | 0.844 | 0.88 (0.61-1.27) | 0.497 |
| Kaplan–Meier estimates were used to calculate cumulative event rates, and Cox proportional hazards models were used to estimate hazard ratios (HRs) with 95% confidence intervals (CIs). Models were adjusted for clustering by trial. Only the first occurrence of each event type was considered for each patient.  CV = cardiovascular; GDMT = guideline-directed medical therapy; MI = myocardial infarction. | | | | | | | | | |

# **Supplementary Table S11.** **Univariable Cox proportional hazards models for clinical outcomes in the pooled randomised cohort (post-discharge outcomes only)**

|  | **GDMT class 4 vs. 0-2** | | **GDMT class 3 vs. 0-2** | | **GDMT class 4 vs. 3** | |
| --- | --- | --- | --- | --- | --- | --- |
|  | **HR (95%CI)** | **P value** | **HR (95%CI)** | **P value** | **HR (95%CI)** | **P value** |
| **At 30 days** |  |  |  |  |  |  |
| Composite of CV death, stroke, or myocardial infarction, n (%) | 0.28 (0.20-0.40) | <0.001 | 0.28 (0.20-0.40) | <0.001 | 1.75 (0.93-3.31) | 0.083 |
| Cardiovascular death, n (%) | 0.23 (0.08-0.66) | 0.006 | 0.23 (0.08-0.66) | 0.004 | 1.42 (0.76-2.64) | 0.267 |
| Myocardial infarction, (%) |  |  |  |  | 2.44 (0.29-20.53) | 0.411 |
| Stroke, n (%) | 0.10 (0.07-0.14) | <0.001 | 0.10 (0.07-0.14) | <0.001 | 0.61 (0.44-0.85) | 0.004 |
| All-cause death, n (%) | 0.23 (0.08-0.66) | 0.006 | 0.23 (0.08-0.66) | 0.004 | 1.42 (0.76-2.64) | 0.267 |
| **At 1 year** |  |  |  |  |  |  |
| Composite of CV death, stroke, or myocardial infarction, n (%) | 0.50 (0.27-0.91) | 0.023 | 0.50 (0.27-0.91) | 0.208 | 0.92 (0.40-2.08) | 0.834 |
| Cardiovascular death, n (%) | 0.30 (0.08-1.09) | 0.068 | 0.30 (0.08-1.09) | 0.433 | 0.63 (0.21-1.89) | 0.412 |
| Myocardial infarction, (%) | 1.48 (0.42-5.20) | 0.543 | 1.48 (0.42-5.20) | 0.130 | 1.89 (0.39-9.16) | 0.427 |
| Stroke, n (%) | 0.42 (0.11-1.66) | 0.217 | 0.42 (0.11-1.66) | 0.912 | 0.46 (0.22-0.95) | 0.037 |
| All-cause death, n (%) | 0.32 (0.11-0.96) | 0.042 | 0.32 (0.11-0.96) | 0.134 | 0.79 (0.37-1.69) | 0.550 |
| **At 5 years** |  |  |  |  |  |  |
| Composite of CV death, stroke, or myocardial infarction, n (%) | 0.47 (0.32-0.71) | <0.001 | 0.47 (0.32-0.71) | 0.010 | 1.07 (0.86-1.33) | 0.554 |
| Cardiovascular death, n (%) | 0.36 (0.18-0.72) | 0.004 | 0.36 (0.18-0.72) | 0.118 | 1.00 (0.54-1.84) | 0.992 |
| Myocardial infarction, (%) | 0.72 (0.56-0.92) | 0.009 | 0.72 (0.56-0.92) | 0.039 | 1.23 (0.91-1.66) | 0.173 |
| Stroke, n (%) | 0.52 (0.29-0.95) | 0.034 | 0.52 (0.29-0.95) | 0.297 | 1.08 (0.50-2.35) | 0.842 |
| All-cause death, n (%) | 0.42 (0.22-0.83) | 0.012 | 0.42 (0.22-0.83) | 0.054 | 1.06 (0.80-1.40) | 0.692 |
| Cox proportional hazards models were used to estimate hazard ratios (HRs) with 95% confidence intervals (CIs). Models were adjusted for clustering by trial.  CV = cardiovascular; GDMT = guideline-directed medical therapy; MI = myocardial infarction. | | | | | | |

# **Supplementary Table S12.** **Sensitivity analysis: clinical outcomes stratified by GDMT class at discharge in** **the real-world PCI registry (only post-discharge outcomes counted)**

|  | **GDMT 4 class at discharge**  **N = 4,606** | **GDMT 3 class at discharge**  **N = 830** | **GDMT 0-2 class at discharge**  **N = 207** | **GDMT class 4 vs. 0-2** | | **GDMT class 3 vs. 0-2** | | **GDMT class 4 vs. 3** | |
| --- | --- | --- | --- | --- | --- | --- | --- | --- | --- |
|  |  |  |  | **Adjusted HR (95%CI)** | **Adjusted P value** | **Adjusted HR (95%CI)** | **Adjusted P value** | **Adjusted HR (95%CI)** | **Adjusted P value** |
| **At 30 days** |  |  |  |  |  |  |  |  |  |
| Composite of CV death, stroke, or myocardial infarction, n (%) | 100 (2.2%) | 34 (4.1%) | 38 (18.4%) | 0.18 (0.11-0.31) | <0.001 | 0.28 (0.15-0.50) | 0.000 | 0.67 (0.41-1.10) | 0.113 |
| Cardiovascular death, n (%) | 59 (1.3%) | 27 (3.3%) | 33 (16.0%) | 0.16 (0.09-0.29) | <0.001 | 0.31 (0.16-0.60) | 0.001 | 0.52 (0.29-0.92) | 0.026 |
| Myocardial infarction, (%) | 35 (0.8%) | 5 (0.6%) | 1 (0.5%) | 0.88 (0.12-6.70) | 0.905 | 0.70 (0.07-6.79) | 0.758 | 1.26 (0.37-4.29) | 0.709 |
| Stroke, n (%) | 13 (0.3%) | 4 (0.5%) | 4 (2.1%) | 0.14 (0.04-0.49) | 0.002 | 0.19 (0.04-0.85) | 0.030 | 0.76 (0.21-2.82) | 0.684 |
| All-cause death, n (%) | 67 (1.5%) | 28 (3.4%) | 36 (17.4%) | 0.16 (0.09-0.28) | <0.001 | 0.27 (0.14-0.51) | <0.001 | 0.60 (0.34-1.04) | 0.067 |
| **At 1 year** |  |  |  |  |  |  |  |  |  |
| Composite of CV death, stroke, or myocardial infarction, n (%) | 225 (4.9%) | 68 (8.2%) | 47 (22.9%) | 0.37 (0.24-0.57) | <0.001 | 0.49 (0.30-0.79) | 0.004 | 0.75 (0.54-1.05) | 0.093 |
| Cardiovascular death, n (%) | 116 (2.53) | 54 (6.5%) | 39 (19.0%) | 0.31 (0.18-0.52) | <0.001 | 0.58 (0.34-1.02) | 0.059 | 0.53 (0.36-0.79) | 0.002 |
| Myocardial infarction, (%) | 87 (1.9%) | 11 (1.4%) | 4 (2.3%) | 2.27 (0.31-16.52) | 0.420 | 1.28 (0.15-10.72) | 0.817 | 1.76 (0.76-4.11) | 0.189 |
| Stroke, n (%) | 40 (0.9%) | 9 (1.1%) | 5 (2.7%) | 0.30 (0.11-0.81) | 0.017 | 0.32 (0.10-1.02) | 0.055 | 0.94 (0.41-2.16) | 0.885 |
| All-cause death, n (%) | 156 (3.4%) | 62 (7.5%) | 48 (23.3%) | 0.29 (0.19-0.45) | <0.001 | 0.44 (0.27-0.71) | 0.001 | 0.66 (0.46-0.95) | 0.024 |
| Kaplan–Meier estimates were used to calculate cumulative event rates, and Cox proportional hazards models were used to estimate hazard ratios (HRs) with 95% confidence intervals (CIs). Only the first occurrence of each event type was considered for each patient.  CV = cardiovascular; GDMT = guideline-directed medical therapy; MI = myocardial infarction. | | | | | | | | | |

# **Supplementary Table S13.** **Univariable Cox proportional hazards models for clinical outcomes in the real-world PCI registry (only post-discharge outcomes counted)**

|  | **GDMT class 4 vs. 0-2** | | **GDMT class 3 vs. 0-2** | | **GDMT class 4 vs. 3** | |
| --- | --- | --- | --- | --- | --- | --- |
|  | **HR (95%CI)** | **P value** | **HR (95%CI)** | **P value** | **HR (95%CI)** | **P value** |
| **At 30 days** |  |  |  |  |  |  |
| Composite of CV death, stroke, or myocardial infarction, n (%) | 0.11 (0.07-0.16) | <0.001 | 0.11 (0.07-0.16) | <0.001 | 0.53 (0.36-0.79) | 0.002 |
| Cardiovascular death, n (%) | 0.07 (0.05-0.11) | <0.001 | 0.07 (0.05-0.11) | <0.001 | 0.39 (0.25-0.62) | <0.001 |
| Myocardial infarction, (%) | 1.46 (0.20-10.67) | 0.708 | 1.46 (0.20-10.67) | 0.888 | 1.25 (0.49-3.20) | 0.637 |
| Stroke, n (%) | 0.11 (0.04-0.35) | <0.001 | 0.11 (0.04-0.35) | 0.037 | 0.49 (0.16-1.54) | 0.223 |
| All-cause death, n (%) | 0.08 (0.05-0.11) | <0.001 | 0.08 (0.05-0.11) | <0.001 | 0.43 (0.28-0.67) | 0.000 |
| **At 1 year** |  |  |  |  |  |  |
| Composite of CV death, stroke, or myocardial infarction, n (%) | 0.19 (0.14-0.26) | <0.001 | 0.19 (0.14-0.26) | <0.001 | 0.59 (0.45-0.77) | <0.001 |
| Cardiovascular death, n (%) | 0.12 (0.08-0.17) | <0.001 | 0.12 (0.08-0.17) | <0.001 | 0.38 (0.28-0.53) | <0.001 |
| Myocardial infarction, (%) | 0.86 (0.31-2.33) | 0.761 | 0.86 (0.31-2.33) | 0.402 | 1.40 (0.75-2.62) | 0.296 |
| Stroke, n (%) | 0.29 (0.11-0.73) | 0.009 | 0.29 (0.11-0.73) | 0.091 | 0.74 (0.36-1.53) | 0.418 |
| All-cause death, n (%) | 0.12 (0.09-0.17) | <0.001 | 0.12 (0.09-0.17) | <0.001 | 0.44 (0.33-0.60) | <0.001 |
| Cox proportional hazards models were used to estimate hazard ratios (HRs) with 95% confidence intervals (CIs).  CV = cardiovascular; GDMT = guideline-directed medical therapy; MI = myocardial infarction. | | | | | | |

# **Supplementary Table S14.** **Univariable Poisson regression models for time-dependent clinical outcomes in the pooled randomised cohort**

|  | **GDMT class 4 vs. 0-2** | | **GDMT class 3 vs. 0-2** | | **GDMT class 4 vs. 3** | |
| --- | --- | --- | --- | --- | --- | --- |
|  | **IRR (95% CI)** | **P value** | **IRR (95% CI)** | **P value** | **IRR (95% CI)** | **P value** |
| Composite of CV death, stroke, or myocardial infarction | 0.72 (0.55-0.94) | 0.017 | 0.72 (0.55-0.94) | 0.004 | 1.14 (0.94-1.37) | 0.172 |
| Cardiovascular death | 0.62 (0.38-1.00) | 0.049 | 0.62 (0.38-1.00) | 0.011 | 1.27 (0.82-1.98) | 0.288 |
| Myocardial infarction | 0.85 (0.62-1.18) | 0.335 | 0.85 (0.62-1.18) | 0.126 | 1.17 (0.93-1.46) | 0.171 |
| Stroke | 0.59 (0.34-1.02) | 0.057 | 0.59 (0.34-1.02) | 0.098 | 0.94 (0.66-1.34) | 0.727 |
| All-cause death | 0.54 (0.37-0.79) | 0.001 | 0.54 (0.37-0.79) | <0.001 | 1.25 (0.87-1.80) | 0.224 |
| Only post-discharge outcomes were included in this analysis. GDMT class was treated as a time-dependent variable and updated at 1 and 2 years if different from the previous period. Incidence rates are expressed per 100 person-years. Poisson regression models with log(person-time) as an offset were used to estimate incidence rate ratios (IRRs) with 95% confidence intervals (CIs). Mixed-effects Poisson regression models with a random effect for patient ID were used to account for within-patient correlation. All event occurrences during each GDMT class period were included in the analysis.  CV = cardiovascular; GDMT = guideline-directed medical therapy; MI = myocardial infarction. | | | | | | |

# **Supplementary Figure S1. Study flowchart**

Flowchart of patient selection for the pooled randomised cohort and the real-world PCI registry.

DES = drug-eluting stent; GDMT = guideline-directed medical therapy; PCI = percutaneous coronary intervention; STEMI = ST-segment elevation myocardial infarction.

# **Supplementary Figure S2. Clinical outcomes stratified by GDMT class at discharge after STEMI in the real-world PCI registry**

Time-to-event curves show the cumulative incidence of clinical outcomes stratified by GDMT class at discharge in the real-world PCI registry.

Abbreviations as in **Figures 1-5**.
